# Outgrowth of erlotinib-resistant subpopulations recapitulated in patient-derived lung tumor spheroids and organoids

**DOI:** 10.1371/journal.pone.0238862

**Published:** 2020-09-08

**Authors:** Malathi Banda, Karen L. McKim, Meagan B. Myers, Masahiro Inoue, Barbara L. Parsons

**Affiliations:** 1 Division of Genetic and Molecular Toxicology, US Food & Drug Administration, National Center for Toxicological Research, Jefferson, Arkansas, United States of America; 2 Osaka Medical Center for Cancer and Cardiovascular Diseases, Osaka, Japan; Virginia Commonwealth University, UNITED STATES

## Abstract

A model that recapitulates development of acquired therapeutic resistance is needed to improve oncology drug development and patient outcomes. To achieve this end, we established methods for the preparation and growth of spheroids from primary human lung adenocarcinomas, including methods to culture, passage, monitor growth, and evaluate changes in mutational profile over time. Primary lung tumor spheroids were cultured in Matrigel^®^ with varying concentrations of erlotinib, a small molecule kinase inhibitor of epidermal growth factor receptor (EGFR) that is ineffective against *KRAS* mutant cells. Subtle changes in spheroid size and number were observed within the first two weeks of culture. Spheroids were cultured for up to 24 weeks, during which time interactions between different cell types, movement, and assembly into heterogeneous organoid structures were documented. Allele-specific competitive blocker PCR (ACB-PCR) was used to quantify low frequency *BRAF* V600E, *KRAS* G12D, *KRAS* G12V, and *PIK3CA* H1047R mutant subpopulations in tumor tissue residue (TR) samples and cultured spheroids. Mutant subpopulations, including multiple mutant subpopulations, were quite prevalent. Twelve examples of mutant enrichment were found in eight of the 14 tumors analyzed, based on the criteria that a statistically-significant increase in mutant fraction was observed relative to both the TR and the no-erlotinib control. Of the mutants quantified in erlotinib-treated cultures, *PIK3CA* H1047 mutant subpopulations increased most often (5/14 tumors), which is consistent with clinical observations. Thus, this ex vivo lung tumor spheroid model replicates the cellular and mutational tumor heterogeneity of human lung adenocarcinomas and can be used to assess the outgrowth of mutant subpopulations. Spheroid cultures with characterized mutant subpopulations could be used to investigate the efficacy of lung cancer combination therapies.

## Introduction

Three-dimensional spheroid and organoid models are currently being evaluated as a means to advance precision oncology [1, 2]. Such models, particularly primary patient-derived spheroid models, more accurately reflect the therapeutic situation than cell lines or mouse xenograft models and, therefore, may be useful in advancing oncology research and personalized treatment of patients [3–5]. Such models are more likely to accurately capture the clonal and subclonal interactions that define tumor phenotype [6]. In particular, primary spheroid or organoid models can be used to address the development of therapeutic resistance, a major roadblock for improving outcomes in patients treated with molecularly-targeted, personalized therapies [7].

Personalized cancer treatments are treatments based on pathologic and genetic characteristics of an individual patient’s tumor, which allow only tumor cells to be targeted, resulting in fewer side effects than are caused by cytotoxic chemotherapy and/or radiation therapy. The IMPACT Study found that across gastrointestinal, gynecologic, breast, melanoma, and lung tumor patients, outcomes were superior in patients whose tumor alterations “matched” MEK/RAF and RET pathways inhibitors than for non-matched therapy [8]. Unfortunately, many studies have demonstrated that the efficacy of targeted therapies is limited by the outgrowth of pre-existing mutant subpopulations that drive resistance to targeted therapies [9–13]. In the well-studied example of colorectal cancer, monotherapy targeting the epidermal growth factor receptor (EGFR) inevitably resulted in the expansion of *KRAS* mutant subpopulations that drive patient relapse [9, 10, 14].

Such observations have generated hypotheses that multi-agent, multi-pathway targeting therapies may be more efficacious than single-agent, targeted therapy [15]. Indeed, combination therapy is a cornerstone of oncology and progress in the area is driving interest in repurposing drugs and targeting multiple tumor pathways at once [7, 16]. Progress in this area has been limited by the lack of human cancer-relevant models to investigate the drivers of therapeutic resistance and whether specific combination therapies abrogate the development of therapeutic resistance. If such models existed, they could be used to compare outcomes of different combination therapies and prioritize them for clinical investigation.

Lung cancer remains a significant public health concern and a challenging disease in terms of personalized cancer treatment. According to NIH SEER statistics (https://seer.cancer.gov/statfacts/html/lungb.html), lung cancer has a five-year survival rate of 20.5% after diagnosis, with 228,820 new cases and 135,720 deaths due to the disease expected in 2020. Currently, lung cancers are screened for actionable mutations, to identify cases suitable for targeted therapies, including those targeting mutations in *EGFR*, *BRAF* and rearrangements in *ALK* or *ROS1* [17, 18]. Tumors with mutations in the *EGFR* (11% of lung cancers) may be treated with erlotinib, gefitinib, afatinib or osimertinib [17]. Tumors with mutations in *BRAF* (7% of lung cancers) may be treated with vemurafenib or dabrafenib, and tumors with rearrangements involving *ALK* and/or *ROS1* (1–2% of lung cancers) may be treated with crizotinib, ceritinib or alectinib [17].

EGFR-targeted therapeutics are reversible-competitive inhibitors of the tyrosine kinase domain, which bind at the adenosine-5’ triphosphate-binding site [19]. Efitinib, erlotinib and afatinib were the first EGFR-TKIs to replace platinum-based chemotherapy as standard first-line treatment for patients with EGFR-mutant disease, particularly for patients with sensitizing *EGFR* exon 19 deletions or the exon 21 L858R mutation [20]. Although the relative merits of EGFR-targeted therapy versus chemotherapy remain uncertain, one large study reported patients who received first-line chemotherapy experienced greater five-year survival rates than those who received first-line EGFR-TKIs, but with detection of resistance causing mutations in the EGFR-targeted treatment groups [21].

The development of resistance to EGFR blockade in the treatment of lung cancer has been investigated for over 10 years, making it one of the best-studied examples of therapeutic resistance [17, 19, 22–26]. Resistance develops in approximately 60% of cases treated with EGFR-targeted therapy, due primarily to the occurrence of a secondary mutation in exon 20, specifically the T790M mutation [20], although L747S, D761Y, T854A mutations cause resistance less frequently [27]. Resistance can also be caused by mutations in *PIK3CA*, *BRAF*, *HER2*, *KRAS*, *STAT3* or *XSL* kinase, or amplifications of *MET*, *EGFR* or *CRKL* [17, 22, 23, 25].

EGFR-targeted therapy can become ineffective, due to the outgrowth of *KRAS* mutant subpopulations. This was first demonstrated for colorectal cancer [9], which has a cancer driver mutational profile similar to lung cancer [28]. Parallel to observations for colorectal cancer, our previous work showed that *KRAS* mutant subpopulations are prevalent in lung adenocarcinomas [29].

Combination therapy/polytherapy is a promising approach for treating lung cancer and abrogating the outgrowth of resistance-causing mutant subpopulations [30]. However, the use of appropriate polytherapies is impeded by the difficulty associated with investigating the efficacy of large numbers of possible drug combinations, given that mutation in multiple genes can drive lung cancer and multiple drugs may be needed to target multiple impacted pathways. To address this concern, we investigated the hypothesis that a primary lung adenocarcinoma model could be developed to provide proof-of-principle regarding the detection and characterization of ex vivo outgrowth of the mutant tumor subpopulations responsible for erlotinib resistance. This approach was chosen for several reasons; 1) the public health impact of lung cancer, 2) lung cancers are frequently treated with inhibitors of EGFR (erlotinib is a first-generation EGFR inhibitor), 3) therapeutic resistance due to EGFR blockade in lung cancer has been well-studied, 4) pre-existing *KRAS* mutant subpopulations have been shown to drive resistance to EGFR blockage in the treatment of colorectal cancer, and 5) allele-specific competitive blocker-PCR (ACB-PCR) analyses have demonstrated that *KRAS* mutant subpopulations are prevalent in lung adenocarcinomas. We utilized ACB-PCR, which can quantify mutant fractions (MFs) as low as 10^−5^ [31–33], to quantify mutant subpopulations before and after culture in the presence or absence of varying doses of erlotinib. We focused on the analysis of *BRAF* V600E, *KRAS* G12D, *KRAS* G12V and *PIK3CA* H1047R because these are known drivers of lung cancer [29] and resistance to EGFR-targeted therapies [17, 23, 24] for which ACB-PCR assays were available [34]. Using this model, we were able to detect the outgrowth of clinically-relevant resistance-driving mutations in culturable material from individual patient tumors, suggesting the model could be used to compare the efficacy of polytherapies and to prioritize them for clinical investigation.

## Materials and methods

This study involved the purchase of anonymous human samples from Bio Options, Brea, CA and the Cooperative Human Tissue Network, Birmingham, AL. The research was reviewed and classified as not human subjects research. Lung adenocarcinoma samples were surgically excised, shipped on wet ice, and received within 24 hours of specimen collection. Specimens (0.25–2.07 grams) were collected from seven male and seven female tissue donors between the ages of 58 and 84. Information on tissue donors and their tumor diagnoses is provided in Table 1.

**Table 1:** Information on tumor tissue donors, sample weights and diagnoses.

Tumor ID	Donor Age, Race, and Gender	Weight (grams)	Specimen Diagnosis
1	84/U/F	1.05	pT2a, moderately differentiated adenocarcinoma, acinar predominant, G2
2	58/W/F	0.54	pT2a, well to moderately differentiated adenocarcinoma
3	67/W/M	0.50	pT2a, adenocarcinoma, solid predominant, with acinar micropapillary
4	67/W/M	0.80	pT2a, poorly differentiated adenocarcinoma, G3, WT EGFR, ROS1 polysomy present
5	64/W/F	2.07	pT1b, pN0 adenocarcinoma, G3 poorly differentiated
6	73/W/F	0.27	pT2b pN1, adenocarcinoma, acinar predominant, G1 well-differentiated
7	64/W/M	0.60	pT2a pN0, adenocarcinoma, acinar predominant, G1 well-differentiated
8	72/W/F	1.39	pT3 pN0 invasive adenocarcinoma, papillary predominant, G2 moderately-differentiated
9	67/C/F	0.70	pT3 pN0 adenocarcinoma, G1 well differentiated
10	70/W/M	0.27	pT2a pN0, adenocarcinoma, solid predominant, G3 poorly-differentiated
11	61/W/M	0.44	pT3, pN0, large cell neuroendocrine carcinoma and adenocarcinoma (WHO category 8013/3, poorly differentiated, G3, EGFR, ROS-1 and KRAS negative)
12	76/B/M	0.80	pT1a pN0 adenocarcinoma, acinar predominant, G1 well-differentiated
13	64/W/M	0.45	pT3 pN0 invasive mucinous adenocarcinoma,
14	83/A/F	0.25	pT3 pN0 adenocarcinoma, acinar predominant, G2 moderately-differentiated

A, Asian; B, Black; F, Female; M, Male; U, Unknown race; W, White

### Tissue processing

**Fig 1:**
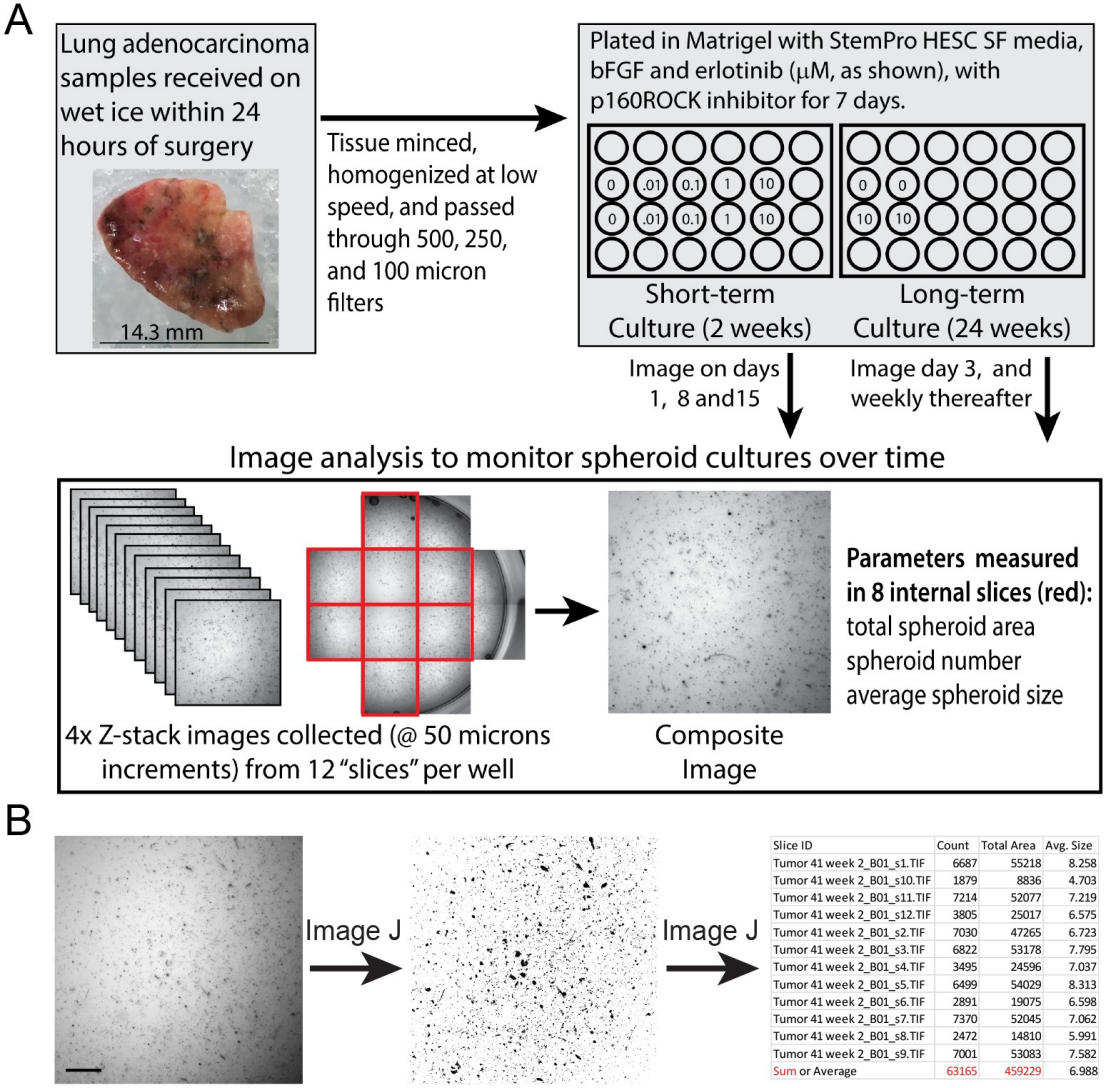
Processing, culture and image analysis of primary lung tumor spheroids. Short- and long-term cultures were plated in Matrigel, and Z-stack images were collected approximately weekly as shown (A). Eight composite images from each well were analyzed using an Image J analyze particle macro to quantify total spheroid area, spheroid number, and average spheroid size (B). The scale bar in (B) is 500 μm.

Procedures for tissue processing and Matrigel culture were based upon methods reported in Kondo et al. and [35] and Endo et al. [36], modified to enable culture of sufficient material for the quantification of mutant subpopulations. Wells of a pre-chilled, 24-well, non-treated tissue culture plate were coated with growth factor reduced Corning^®^ Matrigel^®^ basement membrane matrix (Corning, NY, catalogue number 356230). Specimen processing was performed on ice in a biological safety cabinet as depicted in Fig 1. Acquired portions of excisional biopsies were washed in a 100 cm tissue culture dish using 20 ml Hanks’ balanced salt solution (HBSS), which was then removed by aspiration. Culture media (50 ml) was prepared by combining: 44.89 ml DMEM/F-12, GlutaMAX^™^ supplement, 1 ml Stem Pro hESC Supplement, 3.6 ml 25% bovine serum albumin, 4 μl basic fibroblast growth factor—human recombinant, 3.5 μl 1.43 M 2-mercaptoethanol, and 0.5 ml Penicillin-Streptomycin (10,000 U/mL). All cell culture reagents were Gibco products purchased from ThermoFisher (Waltham, MA). Two ml of freshly prepared media, containing 10 μM Y-27632 dihydrochloride ROCK inhibitor (TOCRIS/Bio-Techne Corporation, Minneapolis, MN), were added to each specimen and minced into 1–2 mm pieces. Minced tissue was transferred to a 13 ml round-bottom, polypropylene centrifuge tube (Sarstedt Inc., Newton, NC) and homogenized for ~1 min on setting 8 using an Omni THq digital tissue homogenizer and a hard tissue Omni tip plastic homogenizing (disposable) probe (Kennesaw, GA). The homogenate was filtered through a 500 μm Corning netwell insert into a 50 ml Falcon tube on ice. Filtrides were collected for subsequent mutational analyses, as pre-culture reference material (tissue residue, TR). Filtrates were subsequently passed through a 250 μm wire mesh in a cup strainer and a 100 μm sterile cell strainer (Fisherbrand^™^, ThermoFisher). Resulting spheroids were collected by centrifugation at 180 g for 5 minutes at 4°C and the supernatant was removed. Cold Matrigel (2.5 ml) containing 10 μM ROCK inhibitor was added to the spheroid pellet and gently triturated using a pre-chilled 10-ml pipette. Next, 200 μl of the suspension was added to wells of a 24-well, flat-bottom culture plate (Corning, Falcon^®^), which had been pre-coated with Matrigel. The plate was left for two hours at room temperature in the biological safety cabinet for the Matrigel to solidify, after which 0.5 ml media was added to each well, based on experimental design described below (Fig 1).

### Culture conditions, duration and erlotinib treatment

Two types of experiments were conducted; (1) using a two-week (15-day) culture period or (2) a 24-week culture period. Erlotinib (erlotinib hydrochloride, Toronto Research Chemicals, Inc., Toronto, Ontario, Canada) was dissolved in DMSO and stored at -20 °C in single use aliquots. Because it was unclear how long the spheroids could be maintained in culture, wells of plated spheroids were cultured initially for two weeks in the media described above, supplemented with 0, 0.01, 0.1, 1, or 10 μM erlotinib (Tumors 1–10, Table 2). Because little change was apparent in most of the erlotinib treatments, next spheroids in replicate wells were cultured in media supplemented with 0 or 10 μM erlotinib for 24 weeks (Tumors 11–14, Table 3). In all cases, media was changed every two to three days. ROCK inhibitor was included in the media during the first seven days of culture. Control (no erlotinib) media included the same amount of DMSO as the 10 μM erlotinib cultures. The long-term cultures grew at different rates, with some showing little to no evidence of growth. Therefore, specimens were passaged differently during the 24-week culture period (cultures from Tumors 12, 13, and 14 were passaged once; Tumor 11 was passaged twice). When passaged, spheroids were recovered as outlined below and re-plated as described above.

**Table 2:** Effects of short-term spheroid culture as assessed by image analyses.

Tumor	Doses of Erlotinib (μM)	Number of Replicate Wells	Significance of Effects Observed in Image J Particle Analysis Data†
**1**	0, 0.001, 0.01, 1 & 10	1	Spheroid Area: TIC*
			Spheroid Number: None
			Average Spheroid Size: TIC***
**2**	0, 0.001, 0.01, 1 & 10	1	Spheroid Area: None
			Spheroid Number: TIC**
			Average Spheroid Size: TIC*
**3**	0, 0.001, 0.01, 1 & 10	2	Spheroid Area: Well*
			Spheroid Number: Well*
			Average Spheroid Size: TIC*, Well*
**4**	0, 0.001, 0.01, 1 & 10	2	Spheroid Area: TIC**
			Spheroid Number: TIC*, Well*
			Average Spheroid Size: TIC***
**5**	0, 0.001, 0.01, 1 & 10	2	Spheroid Area: TIC**
			Spheroid Number: TIC***
			Average Spheroid Size: None
**6**	0, 0.001, 0.01, 1 & 10	2	Spheroid Area: TIC****
			Spheroid Number: TIC****
			Average Spheroid Size: TIC****
**7**	0, 0.001, 0.01, 1 & 10	2	Spheroid Area: TIC****
			Spheroid Number: TIC****
			Average Spheroid Size: TIC***
**8**	0, 0.001, 0.01, 1 & 10	1	Spheroid Area: TIC*
			Spheroid Number: TIC**
			Average Spheroid Size: TIC****
**9**	0, 0.001, 0.01, 1 & 10	2	Spheroid Area: TIC****
			Spheroid Number: TIC****
			Average Spheroid Size: TIC**
**10**	0, 0.001, 0.01, 1 & 10	2	Spheroid Area: TIC****
			Spheroid Number: TIC***
			Average Spheroid Size: TIC*, Well*

^†^ Time in culture, TIC; significance levels are indicated by:

*, P = <0.05;

**, P = <0.01;

***, P <0.001;

****, P <0.0001

**Table 3:** Design and effects observed in long-term spheroid culture as assessed by image analyses.

Tumor	Doses of Erlotinib (μM)	Passage	Number of Replicate Wells	Significance of Effects Observed in Image J Particle Analysis Data†
**11**	0 & 10	Initial	5 (0 & 10 μM)	Spheroid Area: TIC****
				Spheroid Number: None
				Average Spheroid Size: TIC****
		Week 3	5 (0 μM), 11 (10 μM)	Spheroid Area: TIC****, Well****, Interaction ****
				Spheroid Number: TIC****, Well*, Interaction*
				Average Spheroid Size: TIC****, Well****
		Week 10	5 (10 μM)	Spheroid Area: TIC****
				Spheroid Number: TIC**
				Average Spheroid Size: TIC****
**12**	0 & 10	Initial	6 (0 & 10 μM)	Spheroid Area: TIC****, Well****
				Spheroid Number: TIC****, Well****
				Average Spheroid Size: TIC***
		Week 10	6 (0 & 10 μM)	Spheroid Area: Dose**, TIC****, Interaction****
				Spheroid Number: TIC**, Interaction**
				Average Spheroid Size: TIC****, Interaction*
**13**	0 & 10	Initial	5 (0 μM) 6 (10 μM)	Spheroid Area: None
				Spheroid Number: TIC*
				Average Spheroid Size: TIC**
		Week 10	6 (0 & 10 μM)	Spheroid Area: Dose*, TIC****, Interaction****
				Spheroid Number: TIC****, Interaction****
				Average Spheroid Size: TIC****, Well****
**14**	0 & 10	Initial	2 (0 & 10 μM)	Spheroid Area: TIC**, Interaction***, Well****
				Spheroid Number: TIC***, Interaction****, Well****
				Average Spheroid Size: TIC***, Interaction*, Well****
		Week 10	2 (0 & 10 μM)	Spheroid Area: TIC*, Interaction**, Well****
				Spheroid Number: Well*
				Average Spheroid Size: None

^†^ Time in culture, TIC. Unless otherwise stated, repeat measures (RM) two-way ANOVA was performed. Analyses did not assume sphericity. Tumor 11 (11–14 Weeks) 10 μM images were analyzed by RM one-way ANOVA. Tumors 12 & 13 were analyzed using a mix-effects model with Geisser-Greenhouse correction, due to unequal replicates (Tumor 13, 0–10 weeks) or missing images (Tumor 12, two of six replicates missing for week 13). Significance levels are indicated by:

*, P = <0.05;

**, P = <0.01;

***, P <0.001;

****, P <0.0001

### Imaging

Imaging was used to document changes in spheroid cultures and the effect of erlotinib treatment over time. Specifically, a Molecular Devices ImageXpress Micro System and a 4X Plan Apo lens was used to capture images in multiple focal planes (50 μm apart) and Z-stack images were merged into a single composite image (binning = 2). Images corresponding to 12 regions (“slices”) covering the majority of the 2 cm^2^ growth area of each well were collected (see Fig 1). Imaging was performed one, eight and 15 days after plating for the short-term cultures or on Day 3 and weekly thereafter for the 24-week cultures. Also, a Leica MC170 HD microscope and LAS X Leica Application Suite (Buffalo Grove, IL) were used to collect bright field images to document representative changes in spheroid cultures over time.

LAS X 2D image analysis software was used to analyze composite images and define the relationship between pixels (Px) and μm. Specifically, the measurement function of the LAS X 2D image analysis software was used to determine the Molecular Dynamics XL 1.17 megapixel images (1080 x 1080 Px) corresponded to a field 3.5 x 3.5 mm in size. Thus, one Px corresponds to 3.24 μm. Using this conversion, it was determined spheroids in composite images generally ranged from 0.75 to 200 Px in area (8.2 and 1616.7 μm^2^). Batches of composite images were processed and analyzed using Image J 1.50i software (https://imagej.nih.gov/ij/), with analyze particle settings of 0.75–200 Px, to exclude Matrigel bubbles from the analysis. For each field in each well, the Image J analysis output included spheroid count (number), average size (Px), and total area (Px). Only eight of the 12 collected fields were used to quantify effects of time in culture and erlotinib dose on spheroids. As shown in Fig 1, the fields highlighted in red were analyzed (four fields were excluded because only part of the image captured the culture area of the well). Because spheroids were not equivalently distributed within a well, spheroid number and total spheroid area were calculated as the sum of spheroids observed in the eight images of a single well, whereas average spheroid size was calculated as the average of the data from eight images representing a single well.

### Spheroid/organoid recovery, DNA isolation, and re-plating

Media was removed from cultures and Matrigel was washed with 1 ml ice-cold, 1X phosphate-buffered saline, 4mM EDTA, pH 7.4 (PBS, Teknova, Hollister, CA). Another 0.5 ml PBS was added to each well and a rubber policeman was used to scrape the Matrigel from the bottom of each well. A 10-ml pipette was used to transfer the Matrigel to a centrifuge tube, then another 0.5 ml PBS was used to rinse each well and recover any remaining Matrigel. Spheroids were incubated on ice with gentle shaking, and up to an additional 10 mls PBS was added. If all Matrigel had not dissolved, spheroids were collected by centrifugation and the PBS was removed and replaced, with this process repeated until all Matrigel had dissolved. Spheroids were then collected by centrifugation at 180 g for 5 minutes at 4°C and the supernatant removed. Cold Matrigel (2.5 ml) (without ROCK inhibitor) was added and the spheroid pellet was gently triturated using a pre-chilled 10-ml pipette. Then, 200 μl of the suspension was added to wells of a 24-well, flat-bottom culture plate pre-coated with Matrigel and cultured as described above. In the case of some 24-week cultures, different numbers of wells were used for the different specimens, due to differences in initial starting material, differential growth, and the need to retain some material from each passage for DNA isolation (see Table 2).

Spheroids were harvested at the end of the culture period by first dissolving the Matrigel as described above. Next, spheroids were collected by centrifugation (180 g for 5 min at 4°C), the supernatant removed, and DNA in the pellet isolated using Qiagen Allprep DNA/RNA Mini Kit (Qiagen, Germantown, MD). DNA isolation began by homogenization in 350 μl buffer RLT Plus containing β-mercaptoethanol, using an Omni THq digital tissue homogenizer and soft tissue Omni tip plastic homogenizing (disposable) probe. One Qiagen column was used per well, following the manufacturer’s instructions for DNA isolation. DNA isolated from replicate wells was pooled. DNA was also extracted from 120 mg tumor TR (*i*.*e*., the filtride from initial tumor homogenization), by homogenization in 1.4 ml buffer RLT Plus containing β-mercaptoethanol, while using four Qiagen columns per DNA isolation.

All isolated DNA was quantified using the Epoch^™^ spectrophotometer, fitted with a Take3^™^ micro-volume plate (BioTek, Winooski, VT).

### Mutational analyses

ACB-PCR analysis of hotspot cancer driver mutations (CDMs) was a three-step process: 1) a multiplex, first-round PCR of four products was performed, 2) the products were purified, and 3) the four hotspot CDMs were quantified by ACB-PCR. A schematic diagram of the ACB-PCR approach and priming strategy is provided in S1 Fig. High-fidelity PCR was used to concurrently amplify four amplicons encompassing *BRAF* V600E, *EGFR* L858R, *KRAS* G12D/G12V and *PIK3CA* H1047R from 200 ng EcoRI-digested genomic DNA isolated from spheroid/organoid cultures or tumor TR. Each 200 μl PCR reaction mix contained 1.2X *PfuUltra* buffer (12 mM KCl, 12 mM (NH_4_)_2_SO_4_, 24 mM Tris-HCl (pH 8.75), 2.4 mM MgSO_4_, 0.12% Triton X 100, 0.12 mg/ml bovine serum albumin), 1 mM MgCl_2_, 0.4 mM dNTPs, and 30 units of cloned *PfuUltra* Hotstart DNA Polymerase (Agilent Technologies). Primer sequences, their GrCh38 sequence locations and primer concentrations used in the multiplex PCR are provided in Supporting Information S1 Table. Cycling conditions were 94°C for 90 seconds, followed by 35 cycles of 1 min at 94°C, one1 min at 50°C, and 1 min at 72°C, with a final extension of 7 min at 72°C before holding at 4°C. ACB-PCR requires the use of mutant and wild-type and mutant reference standards, which were PCR amplified using cloned plasmid DNA as template, with the PCR optimized to ensure a similar degree of duplication as in the PCR of the genomic DNAs isolated from spheroid cultures. The mutant reference standards (mixed with wild-type) serve as positive controls for mutation detection across a range of MFs.

PCR products generated from the genomic DNA samples or cloned mutant and wild-type plasmids were purified using a Transgenomic WAVE Nucleic Acid Fragment Analysis System (ADS Biotec Inc., Omaha, NE). PCR reactions were applied to a DNASep column and separated as individual peaks by ion pair reverse phase high pressure liquid chromatography (HPLC) using the double stranded multiple fragments application at 4 minutes per 100 base pair. Collection of the four products was based on product length using a threshold collection method for peak identification. The identify of individual peaks was confirmed by comparison with reference DNAs amplified from plasmid DNA containing reference gene segments. All collected PCR products were evaporated to dryness and resuspended in TE buffer (5 mM Tris, 0.5 mM EDTA, pH 7.5). DNA concentrations were measured using the Epoch^™^ spectrophotometer as described above.

ACB-PCR was performed to quantify the mutant fraction (MF, # of mutant alleles/total # of alleles) for four different hotspot CDMs in DNA isolated from each cultured sample and TR. The specific base substitution mutations analyzed were *BRAF* V600E, *KRAS* G12D, *KRAS* G12V, and *PIK3CA* H1047R. The ACB-PCR primers used, with their GrCh38 locations and concentrations employed are provided in Supporting Information S2 Table. Reagents and cycling conditions used in ACB-PCR for the four CDMs are provided in Supporting Information S3 Table.

### Statistical analysis

Statistical analyses of spheroid count and total spheroid area data were based on the sum of the image analysis output for eight fields per well, whereas spheroid size was based on the average across the eight fields. Pre-processing for statistical analysis included setting these Day 1 measurements for short-term cultures and Day 3 measurements for long-term cultures to 1 and expressing subsequent culture duration measurements as proportions of the Day 1 or Day 3 values. Effects of erlotinib treatment and time in culture (TIC) were analyzed by two-way ANOVA for Tumors 1, 2 and 8, for which only a single well was plated. The effects of erlotinib treatment and TIC for all other tumor was performed using repeat measures two-way ANOVA with the Geisser-Greenhouse correction.

Statistical analyses of MF measurements were performed on log-transformed data. MFs below the theoretical limit of ACB-PCR detection (1.37 x 10^−5^ based on the use of 200 ng of input genomic DNA) were set to 1 x 10^−5^. To assess potential mutant enrichment, Mann Whitney rank sum tests were performed on the subset of mutations for which all three replicate MF measurements were greater than those of the corresponding TR or 0 μM erlotinib MF measurements, using one-tailed tests.

For all statistical tests, the threshold for significance was P ≤ 0.05. All statistical analyses were performed using GraphPad Prism 8, Version 8.2.0.

## Results and discussion

Primary lung adenocarcinoma tissue samples, with the pathological diagnoses indicated in Table 1, ranged in mass from 0.25–2.07 grams. After processing, the material is initially in the form of small, round cell aggregates, which are referred to as “spheroids.” Some long-term cultures yielded larger bodies that visually appear to be heterogeneous in size, shape, and cell composition, which are referred to as “organoids.”

At the outset, it was unclear how long spheroids could be maintained in culture. Therefore, following methods development, samples (Tumors 1–10) were cultured for a period of two weeks before harvesting for mutational analyses. Some tumors yielded sufficient material to attempt longer-duration culture and it was ascertained that spheroids could be passaged multiple times and cultured up to 24 weeks. Four long-term cultures (Tumors 11–14) were plated, treated and analyzed for mutation.

**Fig 2:**
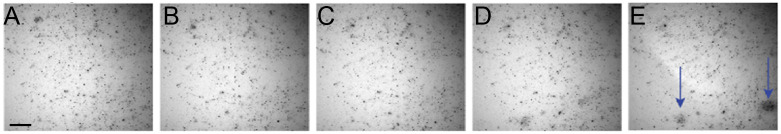
Composite images show only a fraction of spheroids growing over time. Composite images of the same well slice of Tumor 11 collected at (A) Day 3, (B) Week 2, (C) Week 4, (D) Week 6, and (E) Week 8. The scale bar in (B) is 500 μm.

The growth of spheroids was analyzed over time and with respect to erlotinib treatment. Fig 1B shows a representative composite image, background subtraction by Image J, and an example of the output with respect to spheroid quantification. This method reproducibly analyzed the same fields over time (Fig 2) and showed that while most spheroids remain static in culture, specific areas/features of growth were observed.

### Growth characterization of short-term cultures

For the two week cultures only a single well was plated at each dose for Tumors 1, 2, and 8, whereas replicate wells were plated for Tumors 3, 4, 5, 6, 7, 9, and 10. Based upon the eight microscopic fields analyzed per well and including all dose groups, the total spheroid areas varied from 2.48–22.16 μm^2^/well (236,410 to 2,110,439 Px/well). The number of spheroids/well ranged from 32,696 to 474,995, and the average spheroid size per well ranged from 7.35–16.25 μm (4.04–19.76 Px). The coefficient of variation between replicate wells was not negligible (total spheroid area, 15.7%; spheroid number, 14.4%; and average spheroid size, 5.1%). This imprecision likely reflects the technical difficulty in aliquoting homogenized, suspensions of spheroids using a 10 ml pipette (used to minimize losses due to adherence of material during transfer).

To address the problem of different amounts of starting material in replicate wells, parameters measured at Day 1 for each well were set to a value of one and relative measurements at Days 8 and 15 were used to analyze the effects of time in culture and erlotinib dose. The three parameters measured were: 1) the sum of total spheroid area in eight fields per well, 2) the numbers of spheroids in eight fields per well, and 3) the average spheroid size across eight fields per well (Fig 1B). The values of these parameters on Days 8 and 15, relative to Day 1, were analyzed using repeat measure ANOVA. No significant changes in measured parameters were observed related to erlotinib dose in the short-term cultures, but many statistically-significant changes were observed in measured parameters related to time in culture (TIC) (Table 2). Changes in measured parameters for each short-term tumor culture are shown in Supporting Information S2 to S11 Figs.

Combining the analysis of these three parameters provides insight into how processed tumor materials responded to TIC. For example, Tumor 3 showed increases in all three parameters indicating growth over time (S3 Fig). Conversely, Tumors 4 showed decreases in all three parameters, presumed to indicate cell death over time (S4 Fig). Most often the parameters showed mixed results. For example, with increasing time in culture, Tumor 6 showed decreases in spheroid area and number, but an increase in average spheroid size, which was interpreted to indicate that a fraction of spheroids grew and increased in size, while the majority of spheroids disintegrated with increasing TIC (S7 Fig.). Tumors 7 and 9 showed initial decreases in spheroid area and number at day eight, with subsequent increases at day 15, with a concomitant increase in spheroid size, suggesting only a portion of the tumor material was culturable (S7 and S9 Figs). A few statistically-significant “well” effects were observed (Table 2, Tumors 3, 4 and 10), where some samples showed significant differences in measured parameters. Such observations (*e*.*g*., Tumor 3 spheroid area, 0.01 and 0.1 μM erlotinib) suggest the stochastic distribution of rare mutant clones among the culture wells, such that outgrowth occurs in only one or a few wells per sample. Further, it is important to note that the magnitude of changes observed in short-term culture parameters were small, with most of the Day 8 and 15 measurements at ±50% of Day 1 values. A notable exception was average spheroid size for Tumor 6, which increased 4- to 5-fold.

### Growth characterization and properties of long-term cultures

Long-term cultures were first imaged on Day 3, when their parameters were quite similar to the Day 1 short-term cultures. Specifically, for the 0 μM erlotinib wells of Tumors 11–14, total spheroid areas varied from 388,260 to 2,516624 Px/well, the number of spheroids/well ranged from 28,596 to 471,499, and the average spheroid size per well ranged from 5.13–16.38 Px, which is equivalent to an average spheroid diameter between 8.3 and 14.8 μm. Across tumors, the average coefficient of variation between replicate wells was: 10.9% for total spheroid area, 7.0% for spheroid number, and 6.5% for average spheroid size.

**Fig 3:**
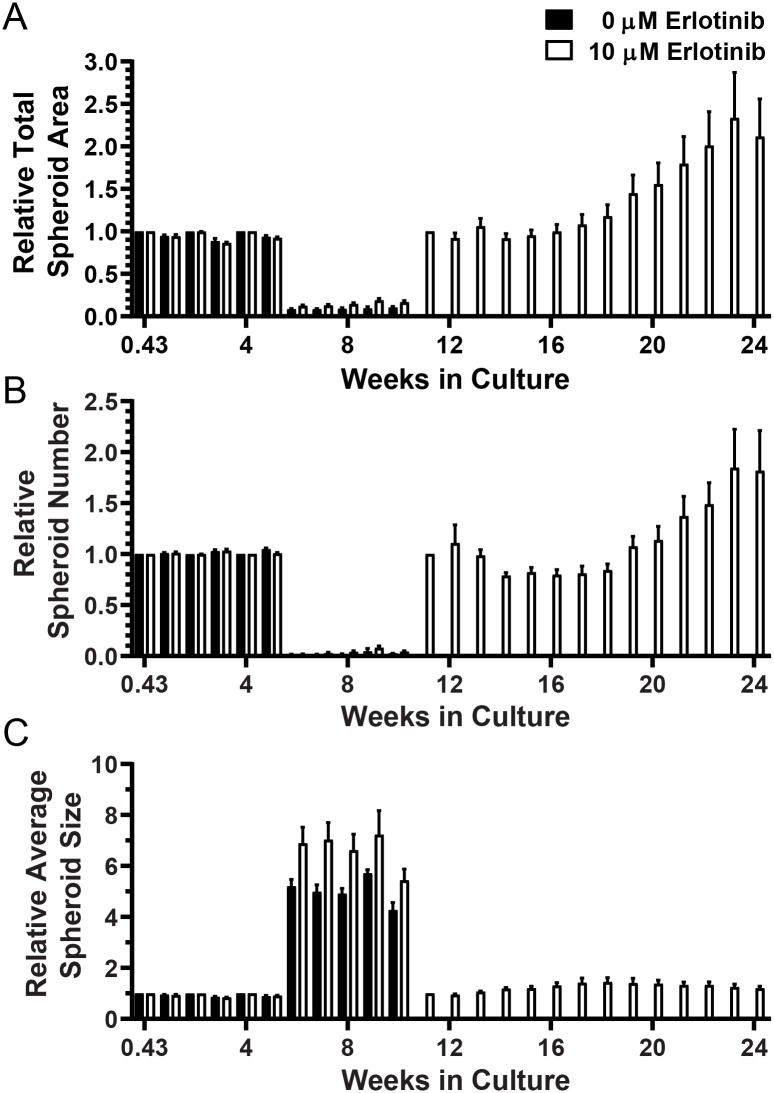
Long-term culture and growth parameters for Tumor 11. (A) Total spheroid area (measured in Px), (B) spheroid number, and (C) average spheroid size measured at Day 3 (0.43 weeks) and after passage at Weeks 3 and 10 were set to 1 for each well and relative repeat measures over time are plotted.

**Fig 4:**
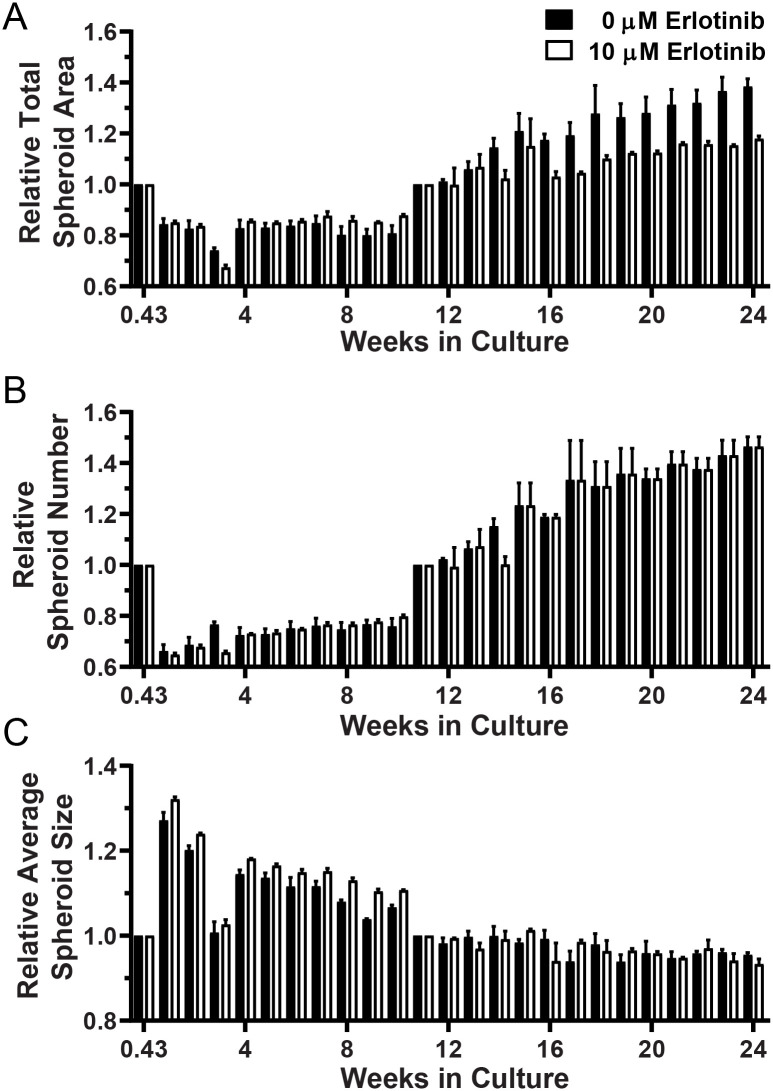
Long-term culture and growth parameters for Tumor 14. (A) Total spheroid area (measured in Px), (B) spheroid number, and (C) average spheroid size measured at Day 3 (0.43 weeks) and after passage at Week 10 were set to 1 for each well and relative repeat measures over time are plotted.

Four cultures (Tumors 11–14) were maintained for 24 weeks. Tumor 11 was passaged after Week 3 and Week 10 imaging, whereas tumors 12, 13, and 14 were passaged only after Week 10 imaging. Recovery of spheroids and re-plating was performed when there appeared to be sufficient material to justify passage or the loss of Matrigel integrity made it necessary. Changes in total spheroid area, spheroid number and average spheroid size relative to that imaged after initial culture or passage are shown for Tumors 11 and 13 in Figs 3 and 4, respectively. The same analyses for Tumors 12 and 14 are provided in S11 and S12 Figs, respectively. After 10 weeks, there was insufficient 0 μM erlotinib material for both DNA isolation and passage, so only the 10 μM erlotinib spheroids were cultured during the 11–24 week period (Fig 3). During the 11–24 week time period, the 10 μM erlotinib Tumor 11 spheroid area and number nearly doubled. Significant changes in culture properties related to TIC were observed for all four long-term cultures (Table 2). Unlike the short-terms cultures, however, significant effects related to erlotinib dose were detected in Tumors 12 and 13 long-term cultures. For example, Fig 4 shows significantly greater growth (as total spheroid area and number) in 0 μM than in 10 μM erlotinib.

**Fig 5:**
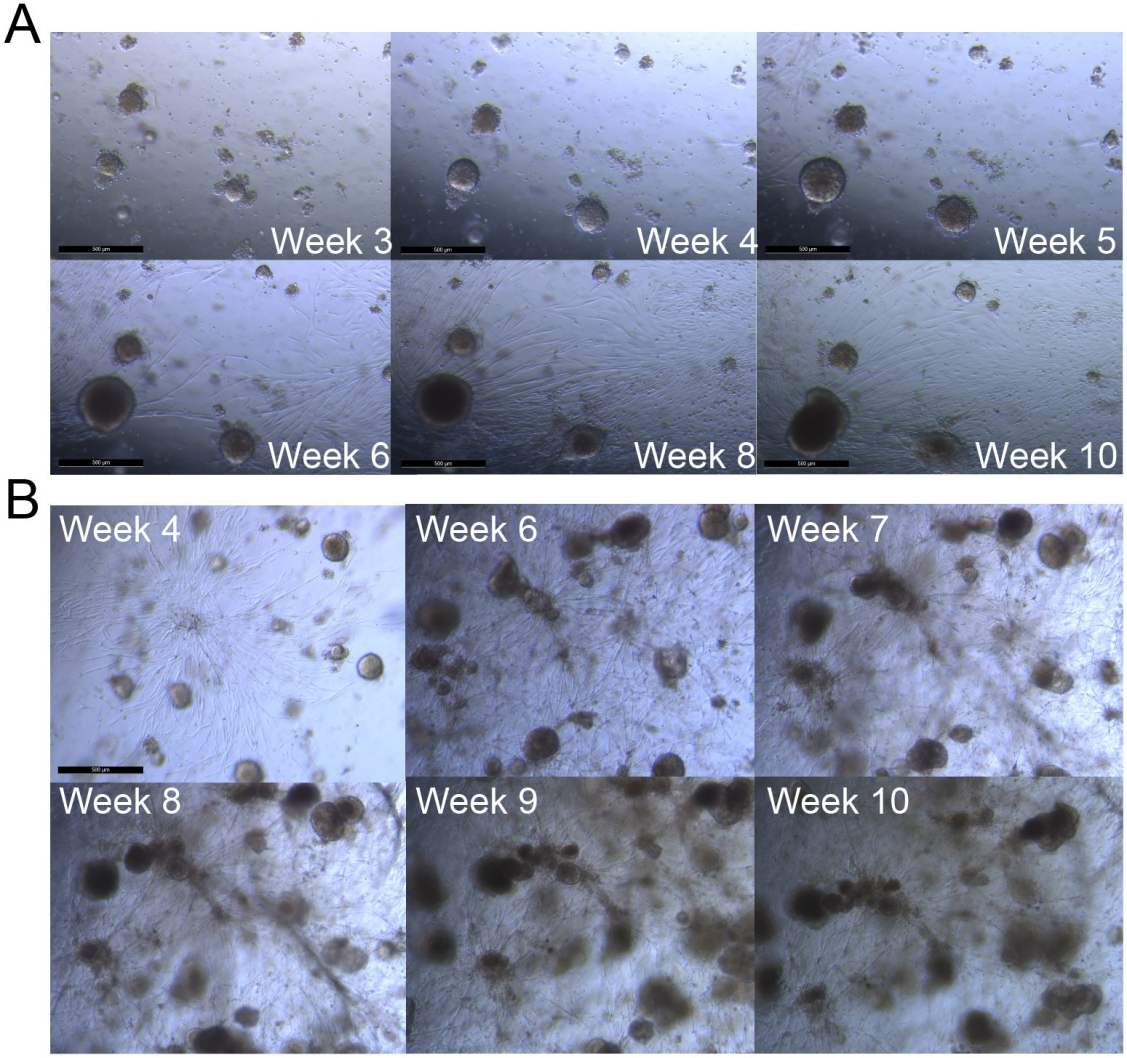
Bright field images of Tumor 11 showing growth of spheroids and fibroblast-like cells over time in 10 μM erlotinib. Sets of images were collected from the same fields of view for two different wells (A and B) between Weeks 3–10 in culture. The scale bars are 500 μm in length.

**Fig 6:**
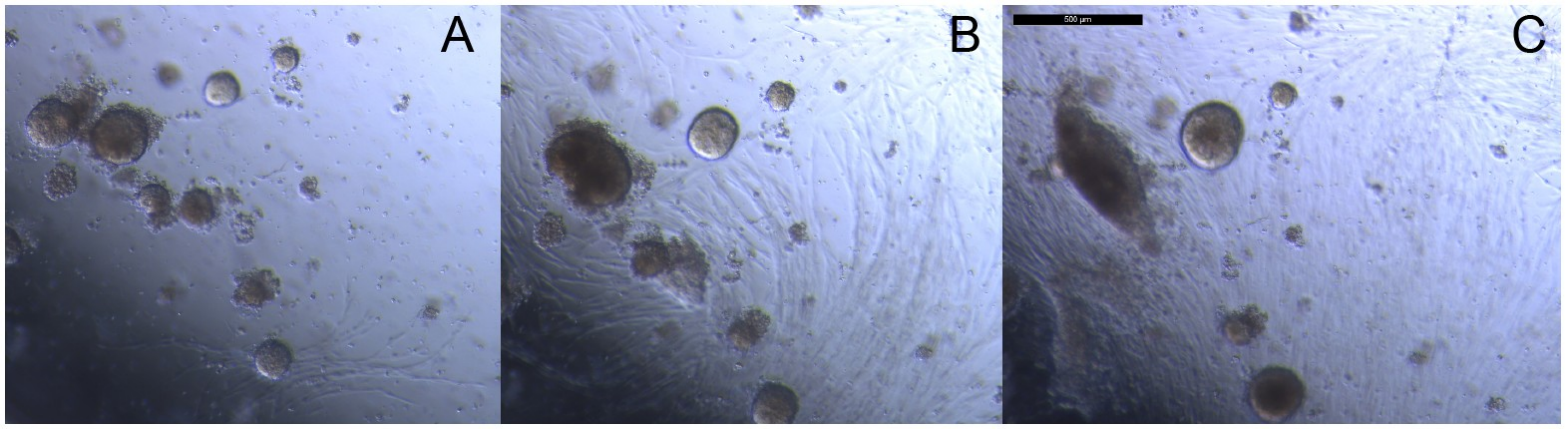
Bright field images of Tumor 11 shows multiple cell types and movement over time. Images collected at (A) Week 4, (B) Week 5, and (C) Week 6. The scale bar (C) is 500 μm.

Several characteristics of spheroid behavior were observed in the long-term cultures. Specifically, observed growth included multiple cell types, movement and aggregation of spheroids, and the development of larger heterogenous bodies, or organoids. The quantitative analyses shown in Tables 1 and 2, Figs 1 and 2 and S2 to S13 Figs indicate that, generally, relatively small changes occurred in the wells overall. However, Fig 5 illustrates the magnitude of changes observed for notable individual spheroids or groups of spheroids. Fig 6 shows the appearance of movement and aggregation of spheroids in Tumor 11 during Weeks 4–6 of culture, as they move along what are presumed to be fibroblast-like cells [37]. This aggregation is apparent in the quantification of average spheroid size for Tumor 11 (see Fig 3). Aggregation of spheroids was observed, particularly following plating, as is evident in the quantification, by increases in average spheroid size with concomitant decreases in spheroid number, without an increase in total spheroid area (Tumors 7 and 8, see S7 and S8 Figs).

**Fig 7:**
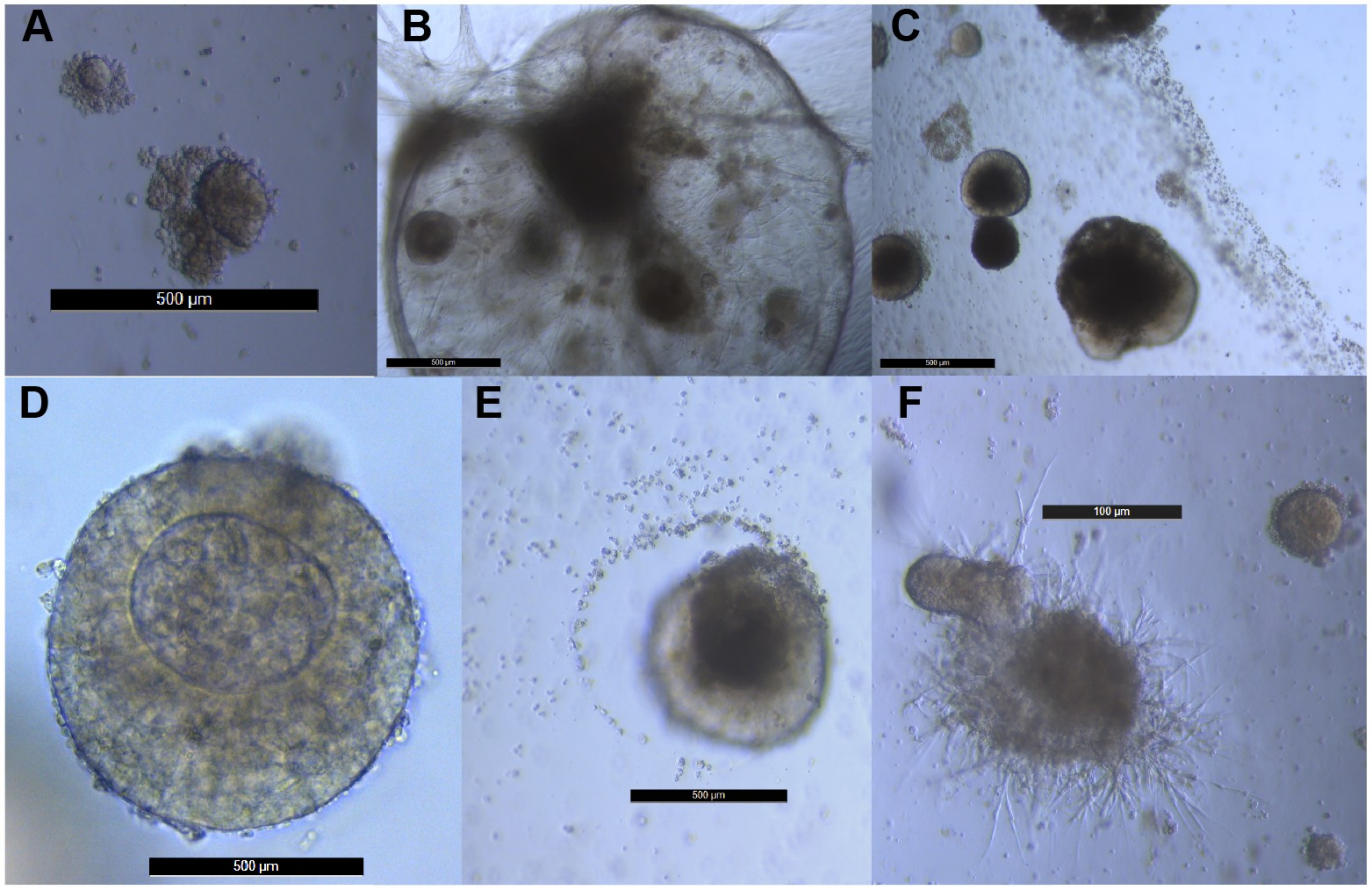
Large and heterogeneous organoids develop in long-term spheroid cultures. The scale bars in panels A-E are 500 μm. The scale bar in panel F is 100 μm.

Large and heterogeneous organoids developed in the long-term cultures, in both the presence and absence of erlotinib. Examples of these are provided in Fig 7. All of the organoids shown in Fig 7 were cultured from Tumor 11, however, organoids were observed in all four long-term cultures and other examples of organoid morphologies observed in Tumors 12, 13 and 14 are shown in S14 Fig. Some organoids attracted smaller spheroids (Fig 7A, 7D and 7F), while others repelled smaller spheroids (Fig 7E). Many of these organoids occurred in fields where fibroblast-like cells and structures were visible (Fig 7B, 7C and 7F). Some organoids were visible without magnification as apparent foci in a culture.

### Analysis of lung cancer and erlotinib-resistance causing mutations

Given the limited amount of DNA that was recovered from spheroid cultures, a multiplex PCR was used to generate amplicons encompassing *BRAF* V600E, *EGFR* L858R, *KRAS* G12D/G12V, and *PIK3CA* H1047R. The input amount of 200 ng is equivalent to 60,000 genomic DNA copies, indicating that ACB-PCR analyses performed would have a theoretical limit of detection of 1.37 x 10^−5^. ACB-PCR analysis of *EGFR* L858R was dropped from the study because a detection sensitivity of 10^−5^ was not achieved reproducibly. For *BRAF* V600E, *KRAS* G12D/G12V, and *PIK3CA* H1047R, generally, three independent ACB-PCR measurement were performed using the first-round Transgenomic WAVE-purified amplicon DNAs as template, with the level of mutation interpreted from a MF standard curve run concurrently in each experiment [38]. In a few cases, insufficient DNA was recovered to perform the first-round PCR and for Tumor 2 only two replicate *KRAS* GAT MF measurements were obtained. The MFs measured in the DNA harvested at the end of the short-term cultures are plotted for each tumor in Supporting Information S2 to S11 Figs, with details regarding any missing datapoints noted in the legends. Similarly, MFs measured in the DNA harvested at the end of the 24-week, long-term culture (and after 10 weeks for Tumor 11) are plotted for each tumor in Figs 3 and 4 and S12 and S13 Figs, with details regarding any missing datapoints noted in the legends. In the context of low-frequency mutational quantification, the total mutational dataset developed from the culture of the original 14 tumors included 962 ACB-PCR MF measurements.

**Fig 8:**
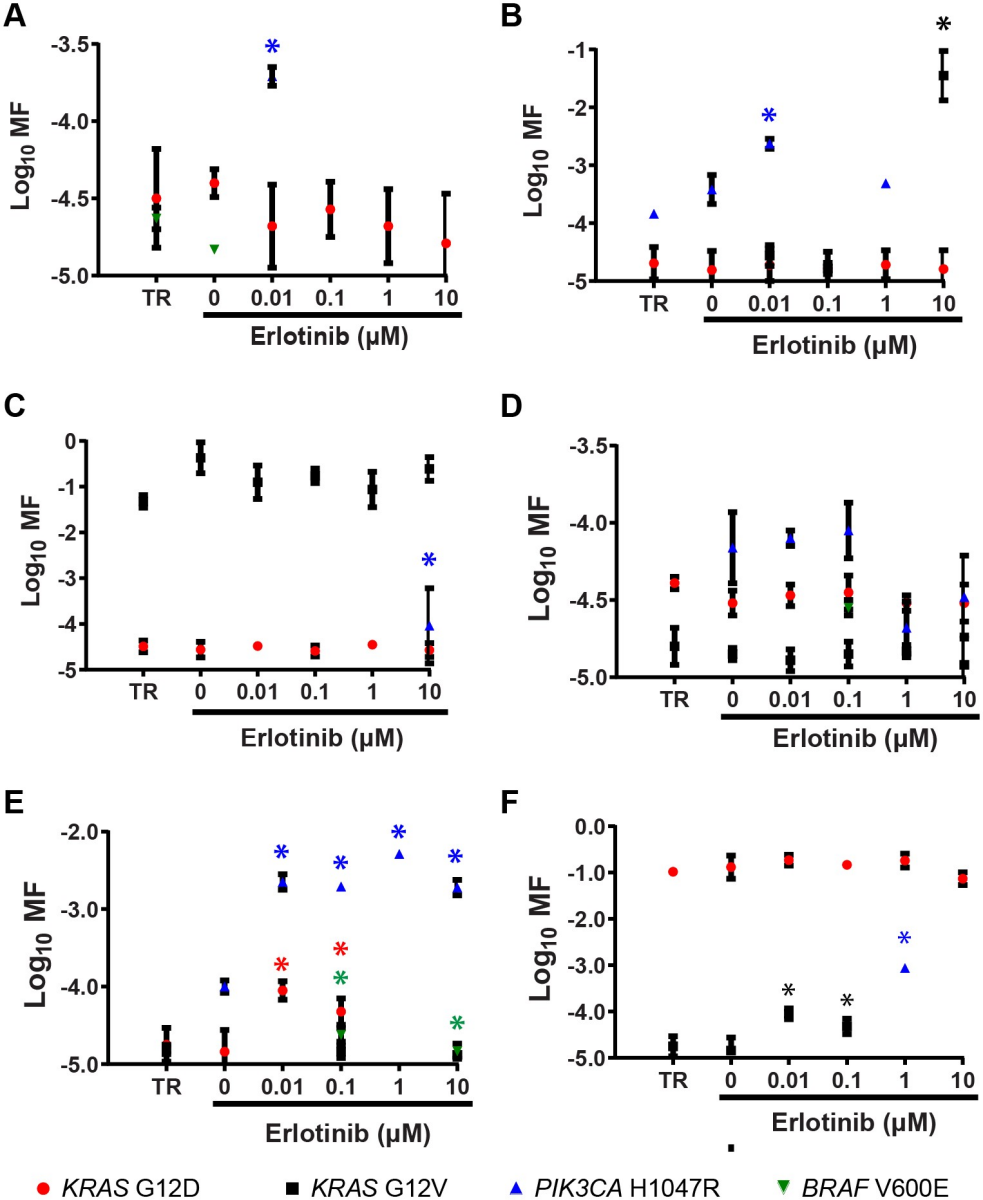
Quantification of mutant subpopulations in DNA harvested from short-term spheroid cultures. Six of the cultures derived from the ten tumors are shown: (A) Tumor 2, (B) Tumor 3, (C) Tumor 4, (D) Tumor 5, (E) Tumor 7, and (F) Tumor 9. Asterisks denote MF measurements significantly greater than that of both TR and the corresponding 0 μM erlotinib culture (one-sided Mann Whitney test, P = 0.0500).

Representative examples of MF measurements obtained from DNAs isolated from the TR or from the spheroid material cultured for two-weeks are given in Fig 8 (data for all tumors are given in S2 to S11 Figs). Mutant subpopulations were prevalent in the cultured tumor samples and, in all but one case, multiple different mutant subpopulations were detected.

Statistical analysis of “enrichment” is complicated by the stochastic nature of carcinogenesis (*i*.*e*., different tumors have different mutational profiles) and inherent difficulties associated with rare event detection (the potential for unequal seeding of a rare mutant may give the appearance of growth in one treatment when it might have grown in any treatment). To address these concerns, to the extent possible, 1) DNA from replicate wells was pooled, and 2) only MFs that were increased relative to both the 0 μM erlotinib and TR MF measurements were tested for statistical significance. Specifically, Mann Whitney rank sum tests were performed on only the subset of mutations for which all three replicate MF measurements were greater than those of the corresponding TR or 0 μM erlotinib MF measurements, which gave *p*-values of 0.0500 in one-tailed tests, using log-transformed and background corrected MFs [specifically, calculated MFs less than the theoretical limit of detection (1.37 x 10^−5^) were set to 1 x 10^−5^]. Cultures that showed a significant enrichment of CDMs in the 2-week and 24-week cultures relative to both the measured TR and 0 μM erlotinib MFs are identified in Table 4. Requiring an increase relative to both 0 μM erlotinib and TR MF measurements reduces the chance that the observed enrichment was due to a stochastic error, because the culture showed a MF that exceeded the non-partitioned input amount (TR). This does not eliminate the possibility that a rare event was seeded in a particular erlotinib-treated well and grew only in that one well. However, the overall prevalence of mutant subpopulations and the fact that enrichment was sometimes observed at multiple erlotinib doses or all doses (Tumor 7) for a given tumor suggests it is unlikely that all of the enrichment reported in Table 4 is due to stochastic effects of associated with mutant well seeding.

**Table 4:** Resistance-causing mutations enriched in erlotinib treated cultures compared to TR and corresponding 0 μM erlotinib control culture.

Tumor Source	Cultures with Increased MF Relative to Both TR and its Corresponding 0 μM Erlotinib Culture	
	Mutation	Erlotinib Dose (μM)
**2**	*PIK3CA* H1047R	0.01
**3**	*KRAS* G12V	10
**3**	*PIK3CA* H1047R	0.01
**4**	*PIK3CA* H1047R	10
**6**	*KRAS* G12V	0.1
**7**	*BRAF* V600E	0.1 & 10
**7**	*KRAS* G12D	0.01 & 0.1
**7**	*PIK3CA* H1047R	0.01, 0.1, 1, & 10
**9**	*KRAS* G12V	0.01 & 0.1
**9**	*PIK3CA* H1047R	1
**12**	*BRAF* V600E	10
**14**	*BRAF* V600E	10

**Fig 9:**
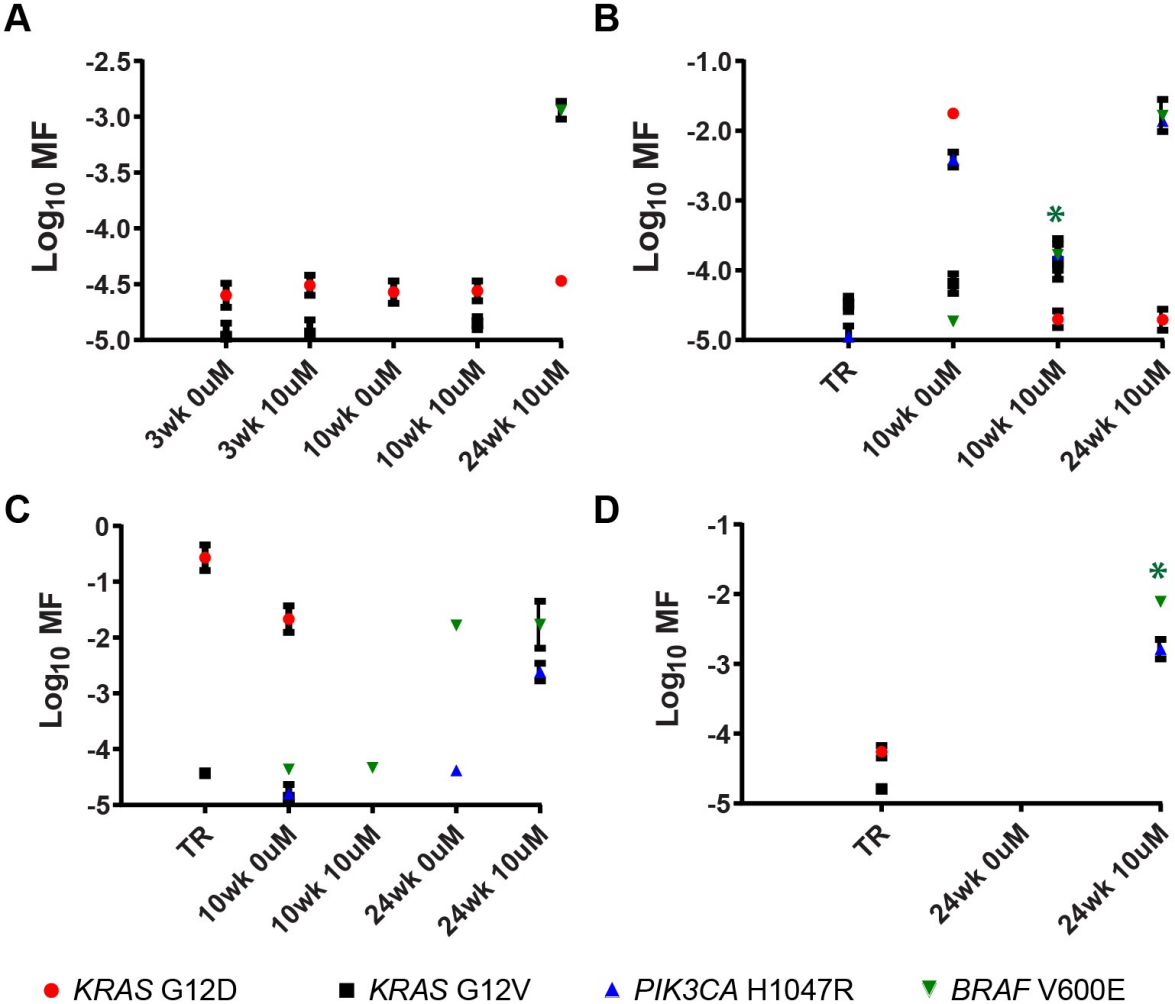
Quantification of mutant subpopulations in DNA harvested from long-term spheroid cultures. Mutant quantification in cultures derived from: (A) Tumor 11, (B) Tumor 12, (C) Tumor 13, (D) and Tumor 14 are shown. Asterisks denote MF measurements significantly greater than that of both TR and its corresponding 0 μM erlotinib culture (one-sided Mann Whitney test, P = 0.0500).

Based upon the defined requirements for concluding that a culture was enriched for mutation(s) (instances denoted by asterisks in Figs 8 and 9, and S2 to S11 Figs), data in Table 4 indicates that ten mutations were enriched in six of the ten short-term cultures, with three enriched in a single mutant and three enriched for multiple mutants. *PIK3CA* H1047R mutants were enriched in five of ten cultures, *KRAS* G12V was enriched in three of ten cultures, with *KRAS* G12D and *BRAF* V600E each enriched in one of the ten short-term cultures. Table 4 shows that *BRAF* V600E was enriched in two of the four long-term cultures. Not all of the amplicons were successfully synthesized from the long-term cultures. Missing datapoints for Tumors 13 and 14 are identified in the legend to Fig 9. Consequently, enrichment for only *BRAF* V600E and *PIK3CA* H1047R were adequately tested for Tumor 13 and only *BRAF* V600E for Tumor 14. Nevertheless, the mutational analyses of short-term and long-term cultures mutational analyses support 12 instances of outgrowth of known resistance-causing CDMs from eight of the tumors analyzed.

## Conclusions

We established 3D-Matrigel culture methods to monitor spheroid growth and noted that the cultures were heterogeneous in appearance and were comprised of multiple cell types. Some cultures exhibited movement and assembly of small spheroids into larger organoids. Some cultures were maintained for up to six months and one culture was frozen in liquid nitrogen and continued to grow after re-plating.

We analyzed four different resistance-causing CDMs in 14 spheroid cultures: *BRAF* V600E, *KRAS* G12D, *KRAS* G12V and *PIK3CA* H1047R. In eight of 14 (57.1%) spheroid cultures, we detected significant erlotinib-associated increases in at least one mutation known to cause therapeutic resistance. Although our previous research showed that *KRAS* mutations are prevalent as subpopulations in lung adenocarcinomas [29], we observed outgrowth of *PIK3CA* H1047R more frequently than *KRAS* mutations, which is consistent with analyses of resistance-associated driver mutations collected in the clinical setting [39].

Multiple CDMs were detected in 13 of 14 spheroid cultures, with six spheroid cultures having all four of the mutations analyzed in at least one of its cultures. Given that only a subsample of possible resistance-driving mutations were analyzed, this highlights the magnitude of the difficulty addressing resistance-causing mutant subpopulations and supports the view that combination therapy is critical for achieving durable responses to molecularly-targeted drug/biologic therapies.

This study highlights some of the challenges of the primary lung tumor organoid approach for investigating combination therapies. Heterogeneity in the tumors makes it a useful model to study clonal interactions within tumors, but also difficult to apply a single prescribed protocol to the continuous culture and passage of all tumor explants. Due to these challenges with culture and passaging and the relatively large amount of DNA needed to detect rare events, the number of different drug and dose combinations that can be investigated at any one time is limited. One potential solution may be to derive panels of spheroid cultures to model tumors with a range of different mutant subpopulations. By replacing the use of tumor cell lines and rodent explant models with characterized human spheroid panels that more closely mimic in vivo conditions, the most effective candidate combinations may then be put forward for clinical investigation, leading to more efficient development of cancer therapeutics. In addition, the primary tumor spheroid culture approach described may be combined with error-corrected next-generation sequencing methods, such as CarcSeq [40] or duplex sequencing [41], to interrogate larger numbers of putative resistance-causing mutations while maximizing the mutational data obtained from limited tumor samples.

## Supporting information

S1 Table Primer sequences, locations and concentrations in first-round multiplex PCR. (DOCX)

S2 Table ACB-PCR primer sequences locations and concentration. (DOCX)

S3 Table Final reaction conditions and cycling conditions for ACB-PCR quantification of mutational targets. (DOCX)

S1 Fig Schematic diagram of the ACB-PCR workflow and priming strategy. ACB-PCR begins by generating equivalent high-fidelity, first-round PCR amplicons from plasmids containing mutant and wild-type reference sequences and the genomic DNA samples being interrogated. In this study, the first-round PCR of genomic DNA samples was a multiplex PCR, amplifying all targets at once. Individual amplicons were purified and quantified. Defined mixtures of mutant and wild-type standards were prepared, with mutant fractions of 10^−1^, 10^−2^, 10^−3^, 10^−4^, 10^−5^ and 0 (wild-type only). Equal copies of standards and unknown products being interrogated were analyzed using the ACB-PCR priming strategy shown (B) and the MFs of unknown samples was calculated from a standard curve based on fluorescent intensity of ACB-PCR products. This figure was adapted from that published in Myers, M.B., K.L. McKim, Y. Wang, M. Banda and Parsons B.L. (2020) ACB-PCR quantification of low-frequency hotspot cancer-driver mutations. In: Molecular Toxicology Protcols, Methods in Molecular Biology, 3rd Ed., P. Keohavong, K.P. Singh, and W. Gao, Eds., Humana Press, Springer Science + Business Media, LLC, New York, NY.
(PDF)

S2 Fig Tumor 1. Quantification of (A) relative total spheroid area, (B) relative spheroid number, and (C) relative average spheroid size, with error bars indicating standard error of the mean. Quantified mutant subpopulations are plotted (D) with error bars indicating standard deviation. Only *KRAS* G12D mutant subpopulations were detected. An example of spheroid culture appearance is provided (E), in which the scale bar is 200 μm in length.
(PDF)

S3 Fig Tumor 2. Quantification of (A) relative total spheroid area, (B) relative spheroid number, and (C) relative average spheroid size, with error bars indicating standard error of the mean. Quantified mutant subpopulations, with error bars indicating standard deviation are shown (D). A significantly larger *PIK3CA* H1047R mutant subpopulation was detected in the 0.01 μM erlotinib spheroid culture as compared to either the 0 μM erlotinib spheroid culture or the Tumor 2 TR (D, one-tailed t-test, P = 0.0500).
(PDF)

S4 Fig Tumor 3. Quantification of (A) relative total spheroid area, (B) relative spheroid number, (C) relative average spheroid size are plotted, with error bars indicating standard error of the mean. Quantified mutant subpopulations are plotted (D), with error bars indicating standard deviation. Significantly larger *PIK3CA* H1047R and *KRAS* G12V mutant subpopulations were quantified in the 0.01 μM and 10 μM erlotinib cultures compared to either the 0 μM erlotinib culture or the Tumor 3 TR, respectively (one-tailed Mann Whitney test, P = 0.0500).
(PDF)

S5 Fig Tumor 4. Quantification of (A) relative total spheroid area, (B) relative spheroid number, and (C) relative average spheroid size, with error bars indicating standard error of the mean. Quantified mutant subpopulations are plotted (D), with error bars indicating standard deviation. Large *KRAS* G12V mutant subpopulations and minor *KRAS* G12D mutant subpopulations were detected in all Tumor 4 cultures. A significantly larger *PIK3CA* H1047R mutant subpopulation was quantified in the 10 μM erlotinib culture as compared to the 0 μM erlotinib culture or Tumor 4 TR (one-tailed t-test, P = 0.0500).
(PDF)

S6 Fig Tumor 5. Quantification of (A) relative total spheroid area, (B) relative spheroid number, and (C) relative average spheroid size, with error bars indicating standard error of the mean. Quantified mutant subpopulations rare plotted (D), with error bars indicating standard deviation. Although subpopulation of all four mutants were present, no erlotinib-treated culture had a significantly larger subpopulation than the 0 μM erlotinib culture and all cultures had *PIK3CA* H1047R mutant subpopulations greater than the Tumor 5 TR (one-tailed Mann Whitney test, P = 0.0500). An example of spheroid culture appearance is provided (E), in which the scale bar = 200 μm.
(PDF)

S7 Fig Tumor 6. Quantification of (A) relative total spheroid area, (B) relative spheroid number, and (C) relative average spheroid size. Quantified mutant subpopulations are plotted (D), with error bars indicating standard deviation. The *KRAS* G12V mutant subpopulation quantified in the 0.1 μM erlotinib culture was significantly greater than that of the 0 μM erlotinib culture or the Tumor 6 TR (one-tailed Mann Whitney test, P = 0.0500). *KRAS* G12D MF measurements were not obtained for the 0 μM erlotinib culture. An example of spheroid culture appearance (E) is provided, in which the scale bar = 500 μm.
(PDF)

S8 Fig Tumor 7. Quantification of (A) relative total spheroid area, (B) relative spheroid number, and (C) relative average spheroid size. Quantified mutant subpopulations are plotted (D), with error bars indicating standard deviation. The *PIK3CA* H1047R mutant subpopulations quantified in the 0.01, 0.1, 1 and 10 μM erlotinib cultures were significantly greater than that of the 0 μM erlotinib culture or the Tumor 6 TR. Similarly, *BRAF* V600E mutant subpopulations in 0.1 and 10 μM erlotinib cultures were significantly greater than that of the 0 μM erlotinib culture or the Tumor 6 TR and *KRAS* G12D mutant subpopulations in 0.01 and 0.1 μM erlotinib cultures were significantly greater than that of the 0 μM erlotinib culture or the Tumor 6 TR (one-tailed Mann Whitney test, P = 0.0500). *KRAS* G12D and *KRAS* G12V MF measurements were not obtained for the 1 μM erlotinib culture. An example of spheroid culture appearance (E) is provided, in which the scale bar = 500 μm.
(PDF)

S9 Fig Tumor 8. Quantification of (A) relative total spheroid area, (B) relative spheroid number, and (C) relative average spheroid size. Quantified mutant subpopulations are plotted (D), with error bars indicating standard deviation. *KRAS* G12V MF measurements were not obtained for the 0 μM erlotinib culture. An example of spheroid culture appearance (E) is provided, in which the scale bar = 500 μm.
(PDF)

S10 Fig Tumor 9. Quantification of (A) relative total spheroid area, (B) relative spheroid number, and (C) relative average spheroid size. Quantified mutant subpopulations are plotted (D), with error bars indicating standard deviation. The *KRAS* G12V mutant subpopulation quantified in the 0.01 and 0.1 μM erlotinib cultures were significantly greater than that of the 0 μM erlotinib culture or the Tumor 6 TR (one-tailed Mann Whitney test, P = 0.0500). The *PIK3CA* H1047R mutant subpopulation quantified in the 0.01 and 0.1 μM erlotinib cultures were significantly greater than those of the 0 μM erlotinib culture or the Tumor 6 TR (one-tailed Mann Whitney test, P = 0.0500). *KRAS* G12V MF measurements were not obtained for the 1 μM erlotinib culture. An example of spheroid culture appearance (E) is provided, in which the scale bar = 500 μm.
(PDF)

S11 Fig Tumor 10. Quantification of (A) relative total spheroid area, (B) relative spheroid number, and (C) relative average spheroid size, with error bars indicating the standard error of the mean. Quantified mutant subpopulations are plotted (D), with error bars indicating standard deviation. Three mutant subpopulations were detected and quantified, but no erlotinib-treated culture showed a significantly larger subpopulation than both the 0 μM erlotinib culture and the Tumor 10 TR (one-tailed Mann Whitney test, P = 0.0500). *PIK3CA* H1047R MF measurements were not obtained for the 0.1 or 1 μM erlotinib cultures. An example of spheroid culture appearance is provided (E), in which the scale bar = 200 μm.
(PDF)

S12 Fig Changes in tumor 12 spheroid culture parameters over time in culture. (A) Total spheroid area, (B) spheroid number, and (C) average spheroid size measured at day 3 (0.43 weeks) after passage at Week 10 were set to one for each well and relative repeat measures over time are plotted. Measurements on Week 13 were considered unreliable and removed because inconsistent repeat measures were obtained for multiple tumors on this day.
(PDF)

S13 Fig Changes in tumor 13 spheroid culture parameters over time in culture. (A) Total spheroid area, (B) spheroid number, and (C) average spheroid size measured at day 3 (0.43 weeks) and passage at Week 10 were set to one for each well and relative repeat measures over time are plotted. Measurements on Week 22 were considered unreliable and removed because inconsistent repeat measures were obtained for multiple tumors on this day.
(PDF)

S14 Fig Additional examples of organoid morphologies. The organoids shown were cultured from Tumor 12 (panel A), Tumor 13 (panels B and F), Tumor 14 (panels C, D and E). The scale bar in panels A, B, D, E and F is 100 μm. The scale bar in panel C is 200 μm. D and E show organoids that appear to have attached to the bottom of the plate and grown primarily as 2D cultures.
(PDF)
