# Supplementary material for: Outgrowth of erlotinib-resistant subpopulations recapitulated in patient-derived lung tumor spheroids and organoids
Source: PLoS One. 2020 Sep 8;15(9):e0238862. doi: 10.1371/journal.pone.0238862 (PMC7478813; doi:10.1371/journal.pone.0238862)
Supplement: S1 Table — (DOCX) [file pone.0238862.s001.docx]

**Table S1. Primer sequences, location and concentrations in first-round multiplex PCR.**

| **Amplicon** | **Primer ID and sequence** | **GRCh38 location** | **Final concentration (nM)** | **Amplicon length (bp)** |
| --- | --- | --- | --- | --- |
| *BRAF* | BRAF-F, 5’-TCATGAAGACCTCACAGTA-3’, | Chr7: 140753383-140753365 | 350 | 153 |
|  | BRAF-R, 5’-AGGGCCAAAAATTTAATCAGT-3’, | Chr7: 140753231-140753251 | 350 |  |
| *EGFR* | EGFRUP, 5’-AACGTACTGGTGAAAACAC-3’ | Chr7: 55191773-55191791 | 350 | 331 |
|  | TR127, 5’-GATAACATCCTCATTCACTG-3’ | Chr7: 55192103-55192084 | 350 |  |
| *KRAS* | RD1, 5’-TTAAGCGTCGATGGAGGAGTT-3’ | Chr12: 25245618-25245598 | 200 | 384 |
|  | RD2, 5’-GTCCTGCACCAGTAATATGC-3’ | Chr12: 25245235-25245254 | 200 |  |
| *PIK3CA* | TR120, 5’-CCAAACTGTTCTTATTACTTATAG-3’ | Chr3: 179234070-179234093 | 500 | 265 |
|  | TR121, 5’-GAAGATCCAATCCATTTTTGT-3’ | Chr3: 179234334-179234314 | 500 |  |
