# Supplementary material for: Outgrowth of erlotinib-resistant subpopulations recapitulated in patient-derived lung tumor spheroids and organoids
Source: PLoS One. 2020 Sep 8;15(9):e0238862. doi: 10.1371/journal.pone.0238862 (PMC7478813; doi:10.1371/journal.pone.0238862)
Supplement: S2 Table — (DOCX) [file pone.0238862.s002.docx]

**Table S2. ACB-PCR primer sequences locations and concentration.**

| **Mutational**  **Target** | **Primer Type** | **Primer ID and sequence (source)†** | **GRCh38 location** | **Concentration (nM)** | **Amplicon length (bp)** |
| --- | --- | --- | --- | --- | --- |
| *BRAF* V600E | Downstream | BRAF DOWN, 5’-/5Biosg/GCCTCAATTCTTACCATCCAC-3’ (IDT) | Chr7: 140753261-140753281 | 400 | 98 |
|  | Mutant-specific | BRAF-MSP, 5’-6-FAM-GGTGATTTTGGTCTAGCTACATA-3’ (IDT) | Chr7: 140753358-140753338 | 400 |  |
|  | Blocker | BRAF-BP, 5’-GGTGATTTTGGTCTAGCTACATT-3’Phos (IDT) | Chr7: 140753358-140753338 | 400 |  |
| *KRAS* G12D | Upstream | P4, 5’-GATTTACCTCTATTGTTGGA-3’ (IDT) | Chr12: 25245267-25245286 | 500 | 103 |
|  | Mutant-specific | MSP-A, 5’-6-FAM-CTTGTGGTAGTTGGAGCTTA-3’ (IDT) | Chr12: 25245369-25245352 | 500 |  |
|  | Blocker | BP-A, 5’-CTTGTGGTAGTTGGAGCTTG-3’Phos (Sigma) | Chr12: 25245369-25245352 | 475 |  |
| *KRAS* G12V | Upstream | P3, 5’-GTTGGATCATATTCGTCCAC-3’ (IDT) | Chr12: 25245281-25245300 | 400 | 89 |
|  | Mutant-specific | MSP-T, 5’-6-FAM-CTTGTGGTAGTTGGAGCTAT-3’ (IDT) | Chr12: 25245369-25245352 | 400 |  |
|  | Blocker | BP-T, 5’-CTTGTGGTAGTTGGAGCTAG-3’Phos (Sigma) | Chr12: 25245369-25245352 | 400 |  |
| *PIK3CA* H1047R | Upstream | TR98, 5’-GATGCTTGGCTCTGGAATGC-3’ (IDT) | Chr3: 179234169-179234188 | 1500 | 211 |
|  | Mutant-specific | TR119, 5’-6-FAM-TGTTGTCCAGCCACCATGTC-3’ (IDT) | Chr3: 179234316-179234299 | 270 |  |
|  | Blocker | TR88, 5’-TGTTGTCCAGCCACCATGTdT (Sigma) | Chr3: 179234316-179234299 | 500 |  |

†Underlined bases are non-complementary bases (with respect to either mutant or wild-type) in the 3’-penultimate position. Source of primers are: IDT, Integrated DNA Technologies; Sigma, Millipore Sigma
