# Supplementary material for: Outgrowth of erlotinib-resistant subpopulations recapitulated in patient-derived lung tumor spheroids and organoids
Source: PLoS One. 2020 Sep 8;15(9):e0238862. doi: 10.1371/journal.pone.0238862 (PMC7478813; doi:10.1371/journal.pone.0238862)
Supplement: S3 Table — (DOCX) [file pone.0238862.s003.docx]

**S3 Table. Final Reaction Conditions and Cycling Conditions for ACB-PCR Quantification of Mutational Targets.**

|  | ***BRAF* V600E** | ***KRAS* G12D** | ***KRAS* G12V** | ***PIK3CA* H1047R** |
| --- | --- | --- | --- | --- |
| **Universal *Taq* Buffer** | 1X | 1X | 1X | 1X |
| **[dNTP] μM** | 80 | 80 | 40 | 40 |
| **[MgCl_2_] mM** | 1.5 | 1.3 | 1.25 | 0.75 |
| **Single-strand DNA binding protein (units)** | 0.66 | none | none | none |
| **PerfectMatch PCR Enhancer (mUnits/ul)** | 1.8 | 2.0 | 2.0 | 1.4 |
| **KlenTaq (uls/50-ul rxn)** | 0.06 | 0.045 | 0.06 | 0.20 |
| **Annealing temperature (◦C)** | 41 | 45 | 41 | 49 |
| **Number of cycles** | 36 | 36 | 36 | 41 |
