# Supplementary material for: Outgrowth of erlotinib-resistant subpopulations recapitulated in patient-derived lung tumor spheroids and organoids
Source: PLoS One. 2020 Sep 8;15(9):e0238862. doi: 10.1371/journal.pone.0238862 (PMC7478813; doi:10.1371/journal.pone.0238862)
Supplement: S1 Fig — ACB-PCR begins by generating equivalent high-fidelity, first-round PCR amplicons from plasmids containing mutant and wild-type reference sequences and the genomic DNA samples being interrogated. In this study, the first-round PCR of genomic DNA samples was a multiplex PCR, amplifying all targets at once. Individual amplicons were purified and quantified. Defined mixtures of mutant and wild-type standards were prepared, with mutant fractions of 10−1, 10−2, 10−3, 10−4, 10−5 and 0 (wild-type only). Equal copies of standards and unknown products being interrogated were analyzed using the ACB-PCR priming strategy shown (B) and the MFs of unknown samples was calculated from a standard curve based on fluorescent intensity of ACB-PCR products. This figure was adapted from that published in Myers, M.B., K.L. McKim, Y. Wang, M. Banda and Parsons B.L. (2020) ACB-PCR quantification of low-frequency hotspot cancer-driver mutations. In: Molecular Toxicology Protcols, Methods in Molecular Biology, 3rd Ed., P. Keohavong, K.P. Singh, and W. Gao, Eds., Humana Press, Springer Science + Business Media, LLC, New York, NY. (PDF) [file pone.0238862.s004.pdf]

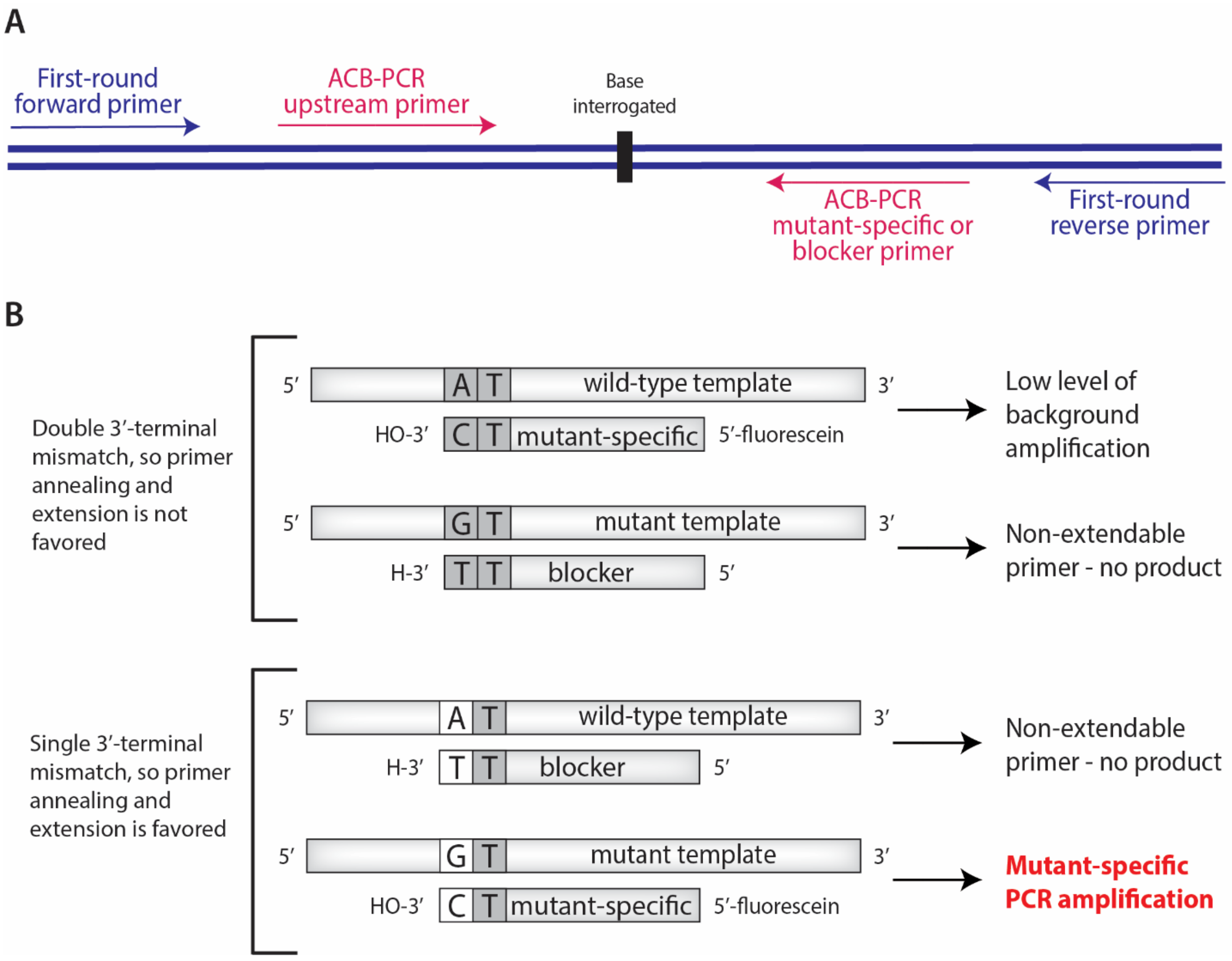

S1 Fig. Schematic diagram of the ACB-PCR workflow and priming strategy.
ACB-PCR begins by generating equivalent high-fidelity, first-round PCR amplicons from plasmids containing mutant and wild-type reference sequences and the genomic DNA samples being interrogated. In this study, the first-round PCR of genomic DNA samples was a multiplex PCR, amplifying all targets at once. Individual amplicons were purified and quantified. Defined mixtures of mutant and wild-type standards were prepared, with mutant fractions of 10^{-1}, 10^{-2}, 10^{-3}, 10^{-4}, 10^{-5} and 0 (wild-type only). Equal copies of standards and unknown products being interrogated were analyzed using the ACB-PCR priming strategy shown (B) and the MFs of unknown samples was calculated from a standard curve based on fluorescent intensity of ACB-PCR products. This figure was adapted from that published in Myers, M.B., K.L. McKim, Y. Wang, M. Banda and Parsons B.L. (2020) ACB-PCR quantification of low-frequency hotspot cancer-driver mutations. In: Molecular Toxicology Protocols, Methods in Molecular Biology, 3rd Ed., P. Keohavong, K.P. Singh, and W. Gao, Eds., Humana Press, Springer Science + Business Media, LLC, New York, NY.
