# Supplementary material for: Outgrowth of erlotinib-resistant subpopulations recapitulated in patient-derived lung tumor spheroids and organoids
Source: PLoS One. 2020 Sep 8;15(9):e0238862. doi: 10.1371/journal.pone.0238862 (PMC7478813; doi:10.1371/journal.pone.0238862)
Supplement: S2 Fig — Quantification of (A) relative total spheroid area, (B) relative spheroid number, and (C) relative average spheroid size, with error bars indicating standard error of the mean. Quantified mutant subpopulations are plotted (D) with error bars indicating standard deviation. Only KRAS G12D mutant subpopulations were detected. An example of spheroid culture appearance is provided (E), in which the scale bar is 200 μm in length. (PDF) [file pone.0238862.s005.pdf]

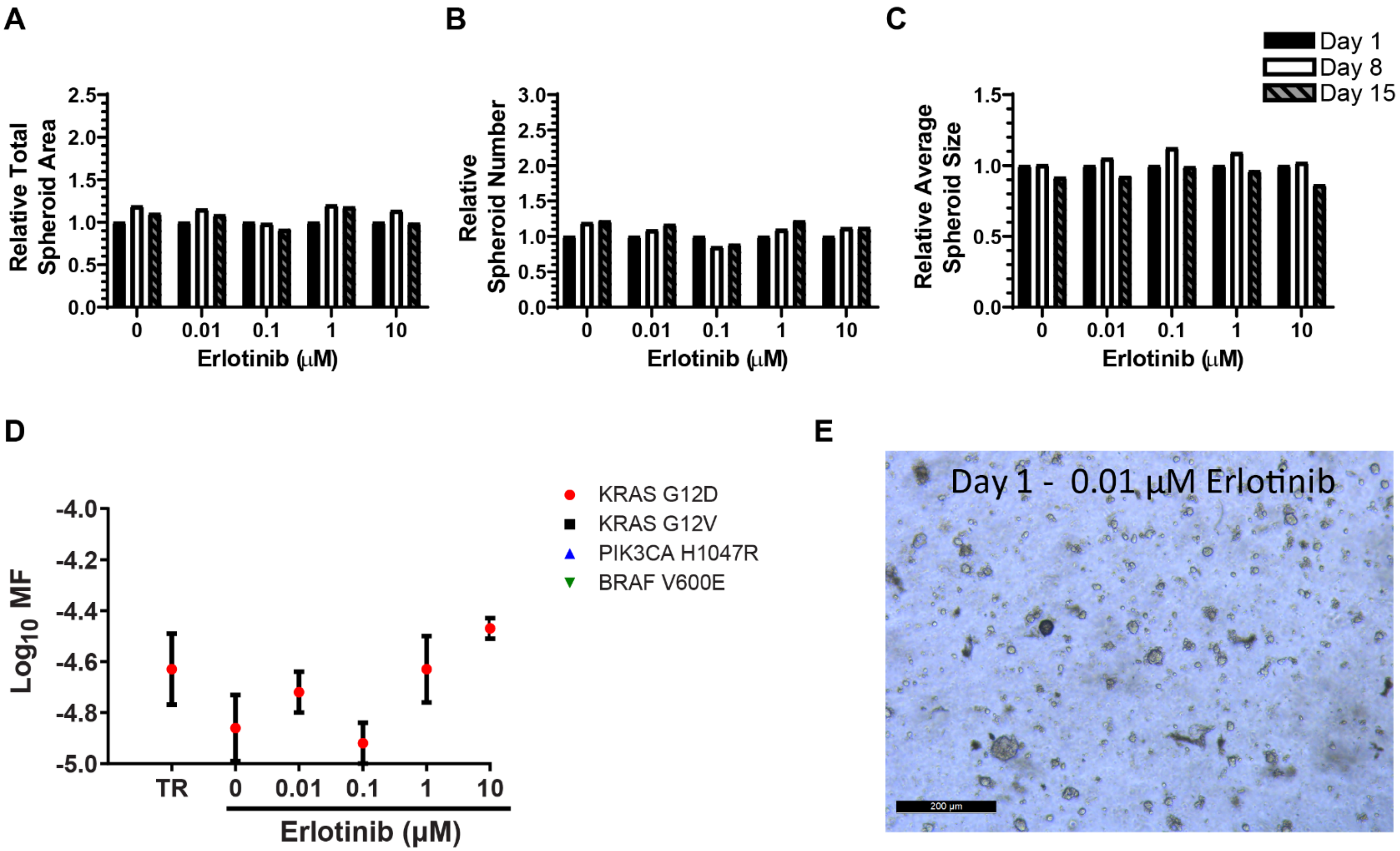

S2 Fig. Tumor 1.

Quantification of (A) relative total spheroid area, (B) relative spheroid number, and (C) relative average spheroid size, with error bars indicating standard error of the mean. Quantified mutant subpopulations are plotted (D) with error bars indicating standard deviation. Only *KRAS* G12D mutant subpopulations were detected. An example of spheroid culture appearance is provided (E), in which the scale bar is 200 µm in length.
