# Supplementary material for: Outgrowth of erlotinib-resistant subpopulations recapitulated in patient-derived lung tumor spheroids and organoids
Source: PLoS One. 2020 Sep 8;15(9):e0238862. doi: 10.1371/journal.pone.0238862 (PMC7478813; doi:10.1371/journal.pone.0238862)
Supplement: S3 Fig — Quantification of (A) relative total spheroid area, (B) relative spheroid number, and (C) relative average spheroid size, with error bars indicating standard error of the mean. Quantified mutant subpopulations, with error bars indicating standard deviation are shown (D). A significantly larger PIK3CA H1047R mutant subpopulation was detected in the 0.01 μM erlotinib spheroid culture as compared to either the 0 μM erlotinib spheroid culture or the Tumor 2 TR (D, one-tailed t-test, P = 0.0500). (PDF) [file pone.0238862.s006.pdf]

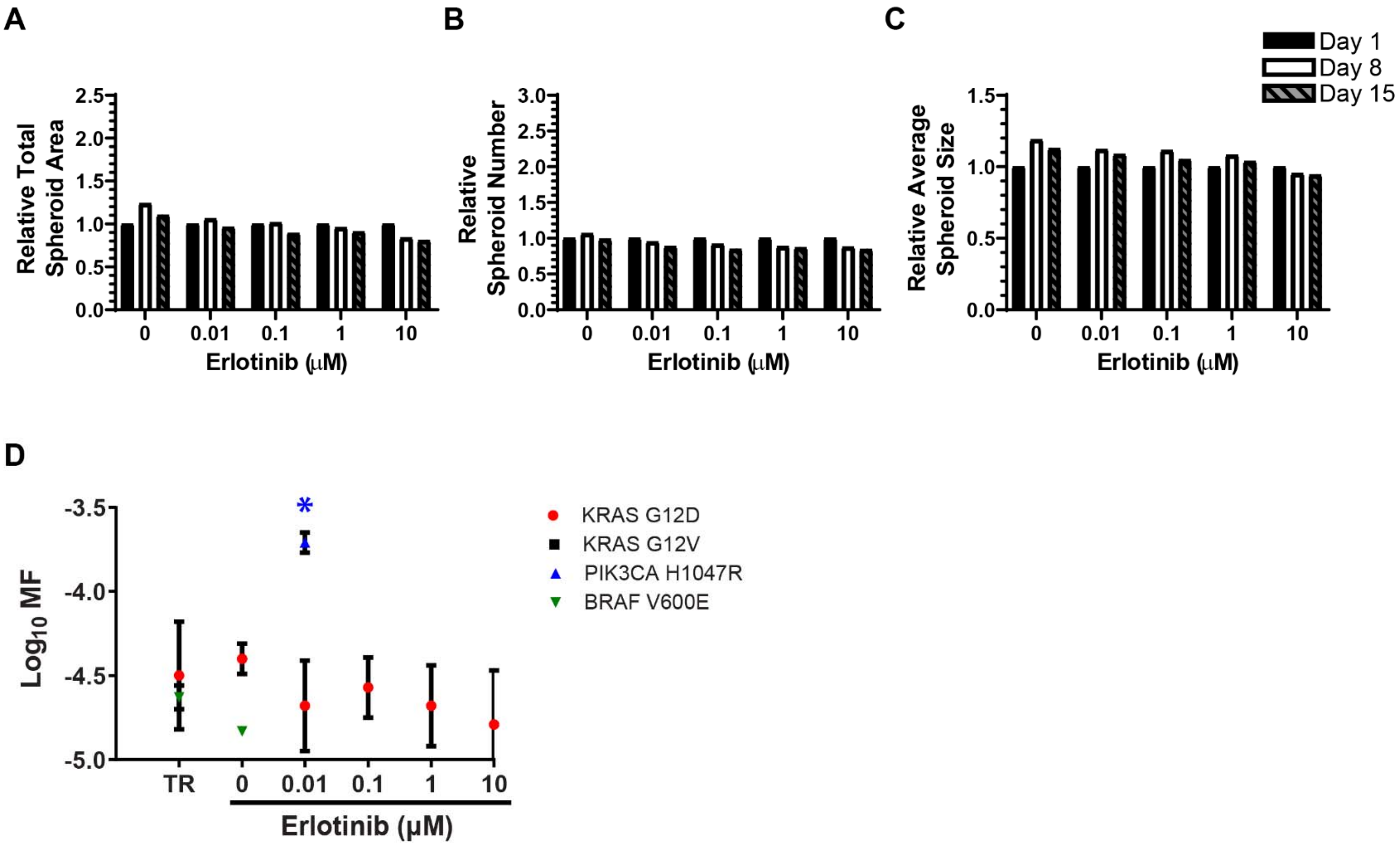

S3 Fig. Tumor 2.

Quantification of (A) relative total spheroid area, (B) relative spheroid number, and (C) relative average spheroid size, with error bars indicating standard error of the mean. Quantified mutant subpopulations, with error bars indicating standard deviation are shown (D). A significantly larger *PIK3CA* H1047R mutant subpopulation was detected in the 0.01 µM erlotinib spheroid culture as compared to either the 0 µM erlotinib spheroid culture or the Tumor 2 TR (D, one-tailed t-test, P = 0.0500).
