# Supplementary material for: Outgrowth of erlotinib-resistant subpopulations recapitulated in patient-derived lung tumor spheroids and organoids
Source: PLoS One. 2020 Sep 8;15(9):e0238862. doi: 10.1371/journal.pone.0238862 (PMC7478813; doi:10.1371/journal.pone.0238862)
Supplement: S4 Fig — Quantification of (A) relative total spheroid area, (B) relative spheroid number, (C) relative average spheroid size are plotted, with error bars indicating standard error of the mean. Quantified mutant subpopulations are plotted (D), with error bars indicating standard deviation. Significantly larger PIK3CA H1047R and KRAS G12V mutant subpopulations were quantified in the 0.01 μM and 10 μM erlotinib cultures compared to either the 0 μM erlotinib culture or the Tumor 3 TR, respectively (one-tailed Mann Whitney test, P = 0.0500). (PDF) [file pone.0238862.s007.pdf]

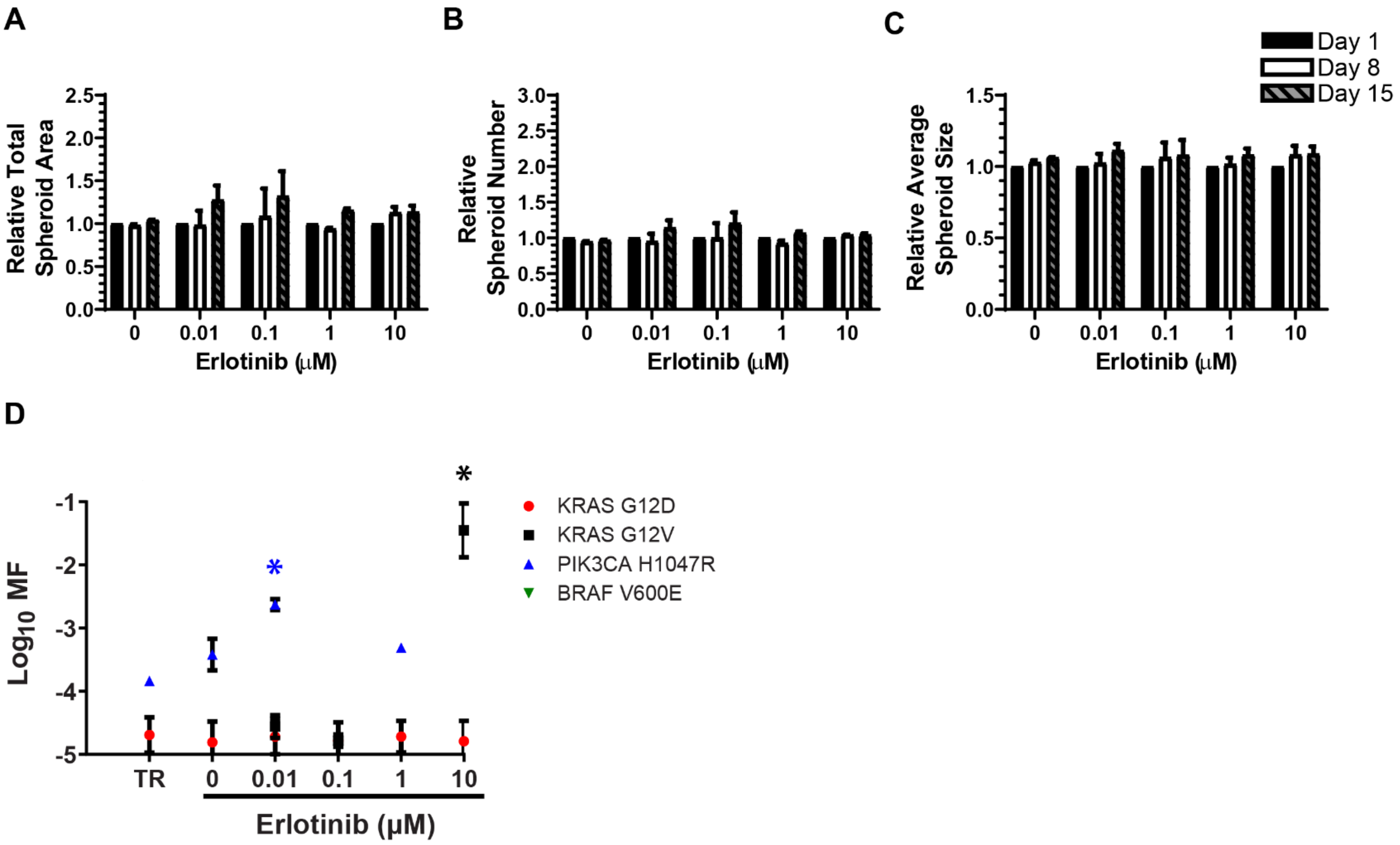

S4 Fig. Tumor 3.

Quantification of (A) relative total spheroid area, (B) relative spheroid number, (C) relative average spheroid size are plotted, with error bars indicating standard error of the mean. Quantified mutant subpopulations are plotted (D), with error bars indicating standard deviation. Significantly larger *PIK3CA* H1047R and *KRAS* G12V mutant subpopulations were quantified in the 0.01 μM and 10 μM erlotinib cultures compared to either the 0 μM erlotinib culture or the Tumor 3 TR, respectively (one-tailed Mann Whitney test, P = 0.0500).
