# Supplementary material for: Outgrowth of erlotinib-resistant subpopulations recapitulated in patient-derived lung tumor spheroids and organoids
Source: PLoS One. 2020 Sep 8;15(9):e0238862. doi: 10.1371/journal.pone.0238862 (PMC7478813; doi:10.1371/journal.pone.0238862)
Supplement: S5 Fig — Quantification of (A) relative total spheroid area, (B) relative spheroid number, and (C) relative average spheroid size, with error bars indicating standard error of the mean. Quantified mutant subpopulations are plotted (D), with error bars indicating standard deviation. Large KRAS G12V mutant subpopulations and minor KRAS G12D mutant subpopulations were detected in all Tumor 4 cultures. A significantly larger PIK3CA H1047R mutant subpopulation was quantified in the 10 μM erlotinib culture as compared to the 0 μM erlotinib culture or Tumor 4 TR (one-tailed t-test, P = 0.0500). (PDF) [file pone.0238862.s008.pdf]

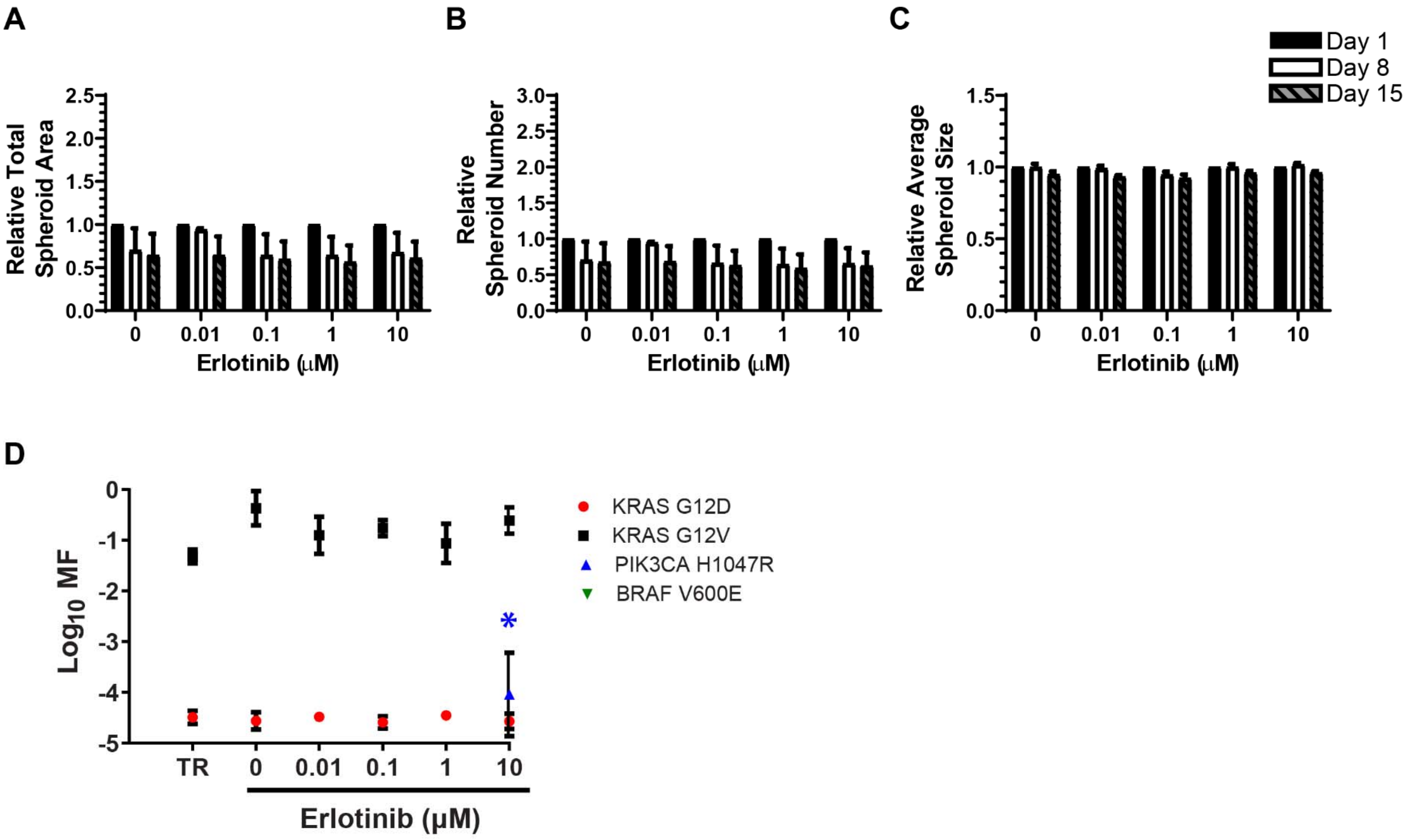

S5 Fig. Tumor 4.

Quantification of (A) relative total spheroid area, (B) relative spheroid number, and (C) relative average spheroid size, with error bars indicating standard error of the mean. Quantified mutant subpopulations are plotted (D), with error bars indicating standard deviation. Large *KRAS* G12V mutant subpopulations and minor *KRAS* G12D mutant subpopulations were detected in all Tumor 4 cultures. A significantly larger *PIK3CA* H1047R mutant subpopulation was quantified in the 10 μM erlotinib culture as compared to the 0 μM erlotinib culture or Tumor 4 TR (one-tailed t-test, $P = 0.0500$).
