# Supplementary material for: Outgrowth of erlotinib-resistant subpopulations recapitulated in patient-derived lung tumor spheroids and organoids
Source: PLoS One. 2020 Sep 8;15(9):e0238862. doi: 10.1371/journal.pone.0238862 (PMC7478813; doi:10.1371/journal.pone.0238862)
Supplement: S6 Fig — Quantification of (A) relative total spheroid area, (B) relative spheroid number, and (C) relative average spheroid size, with error bars indicating standard error of the mean. Quantified mutant subpopulations rare plotted (D), with error bars indicating standard deviation. Although subpopulation of all four mutants were present, no erlotinib-treated culture had a significantly larger subpopulation than the 0 μM erlotinib culture and all cultures had PIK3CA H1047R mutant subpopulations greater than the Tumor 5 TR (one-tailed Mann Whitney test, P = 0.0500). An example of spheroid culture appearance is provided (E), in which the scale bar = 200 μm. (PDF) [file pone.0238862.s009.pdf]

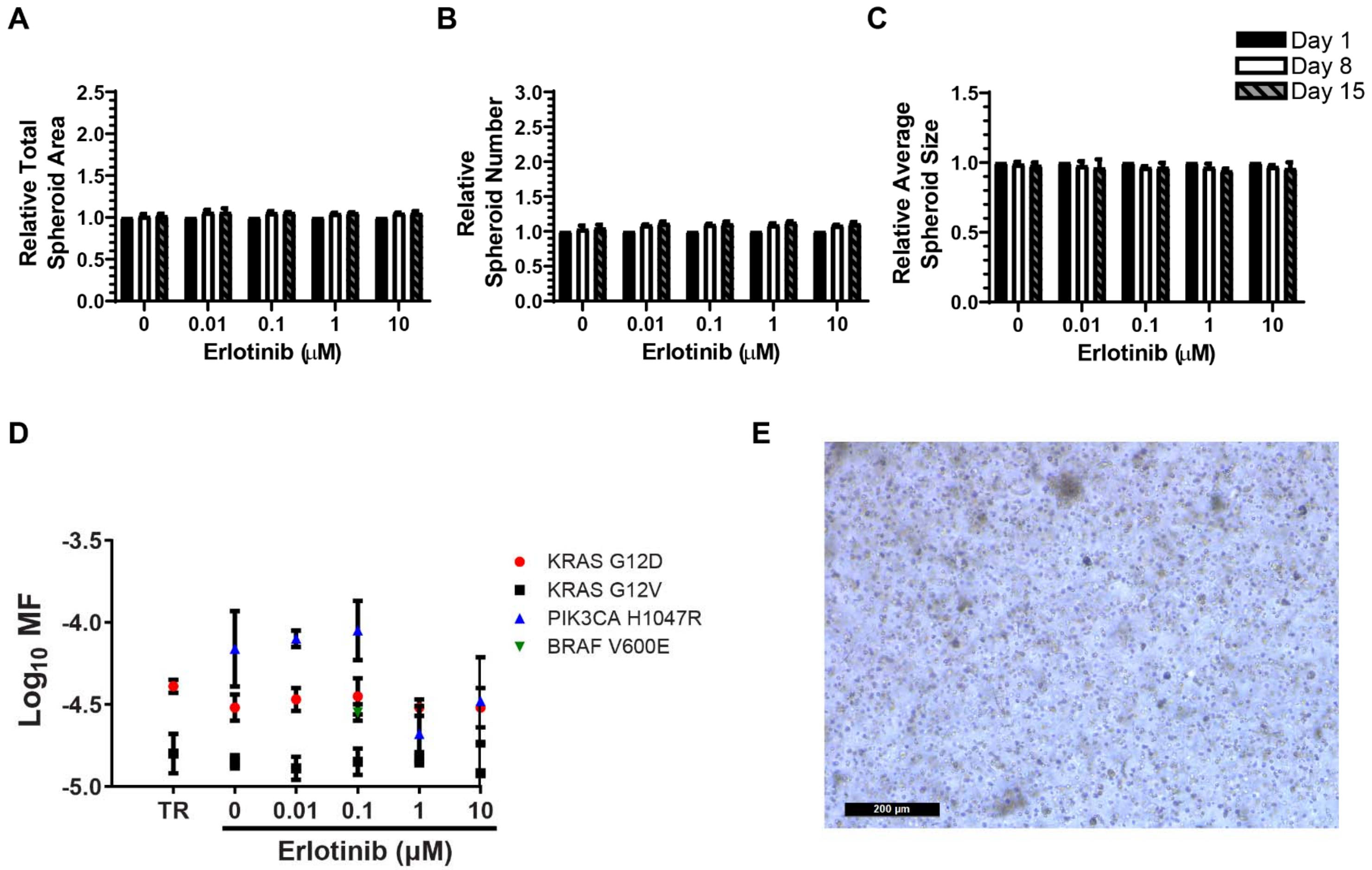

S6 Fig. Tumor 5.

Quantification of (A) relative total spheroid area, (B) relative spheroid number, and (C) relative average spheroid size, with error bars indicating standard error of the mean. Quantified mutant subpopulations rare plotted (D), with error bars indicating standard deviation. Although subpopulation of all four mutants were present, no erlotinib-treated culture had a significantly larger subpopulation than the 0 µM erlotinib culture and all cultures had *PIK3CA* H1047R mutant subpopulations greater than the Tumor 5 TR (one-tailed Mann Whitney test, $P = 0.0500$). An example of spheroid culture appearance is provided (E), in which the scale bar = 200 µm.
