# Supplementary material for: Outgrowth of erlotinib-resistant subpopulations recapitulated in patient-derived lung tumor spheroids and organoids
Source: PLoS One. 2020 Sep 8;15(9):e0238862. doi: 10.1371/journal.pone.0238862 (PMC7478813; doi:10.1371/journal.pone.0238862)
Supplement: S8 Fig — Quantification of (A) relative total spheroid area, (B) relative spheroid number, and (C) relative average spheroid size. Quantified mutant subpopulations are plotted (D), with error bars indicating standard deviation. The PIK3CA H1047R mutant subpopulations quantified in the 0.01, 0.1, 1 and 10 μM erlotinib cultures were significantly greater than that of the 0 μM erlotinib culture or the Tumor 6 TR. Similarly, BRAF V600E mutant subpopulations in 0.1 and 10 μM erlotinib cultures were significantly greater than that of the 0 μM erlotinib culture or the Tumor 6 TR and KRAS G12D mutant subpopulations in 0.01 and 0.1 μM erlotinib cultures were significantly greater than that of the 0 μM erlotinib culture or the Tumor 6 TR (one-tailed Mann Whitney test, P = 0.0500). KRAS G12D and KRAS G12V MF measurements were not obtained for the 1 μM erlotinib culture. An example of spheroid culture appearance (E) is provided, in which the scale bar = 500 μm. (PDF) [file pone.0238862.s011.pdf]

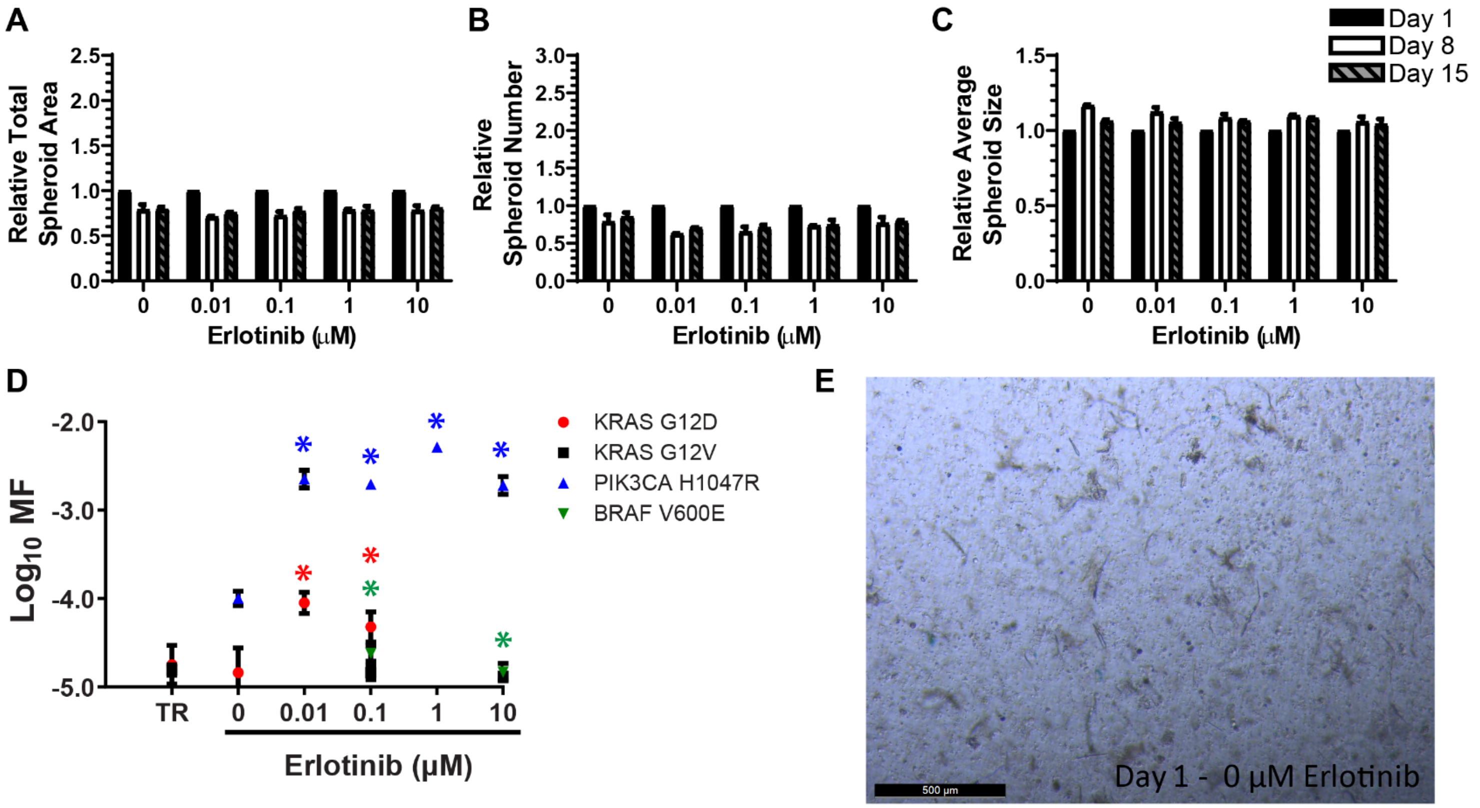

S8 Fig. Tumor 7.

Quantification of (A) relative total spheroid area, (B) relative spheroid number, and (C) relative average spheroid size. Quantified mutant subpopulations are plotted (D), with error bars indicating standard deviation. The *PIK3CA* H1047R mutant subpopulations quantified in the 0.01, 0.1, 1 and 10 µM erlotinib cultures were significantly greater than that of the 0 µM erlotinib culture or the Tumor 6 TR. Similarly, *BRAF* V600E mutant subpopulations in 0.1 and 10 µM erlotinib cultures were significantly greater than that of the 0 µM erlotinib culture or the Tumor 6 TR and *KRAS* G12D mutant subpopulations in 0.01 and 0.1 µM erlotinib cultures were significantly greater than that of the 0 µM erlotinib culture or the Tumor 6 TR (one-tailed Mann Whitney test, P = 0.0500). *KRAS* G12D and *KRAS* G12V MF measurements were not obtained for the 1 µM erlotinib culture. An example of spheroid culture appearance (E) is provided, in which the scale bar = 500 µm.
