# Supplementary material for: Outgrowth of erlotinib-resistant subpopulations recapitulated in patient-derived lung tumor spheroids and organoids
Source: PLoS One. 2020 Sep 8;15(9):e0238862. doi: 10.1371/journal.pone.0238862 (PMC7478813; doi:10.1371/journal.pone.0238862)
Supplement: S9 Fig — Quantification of (A) relative total spheroid area, (B) relative spheroid number, and (C) relative average spheroid size. Quantified mutant subpopulations are plotted (D), with error bars indicating standard deviation. KRAS G12V MF measurements were not obtained for the 0 μM erlotinib culture. An example of spheroid culture appearance (E) is provided, in which the scale bar = 500 μm. (PDF) [file pone.0238862.s012.pdf]

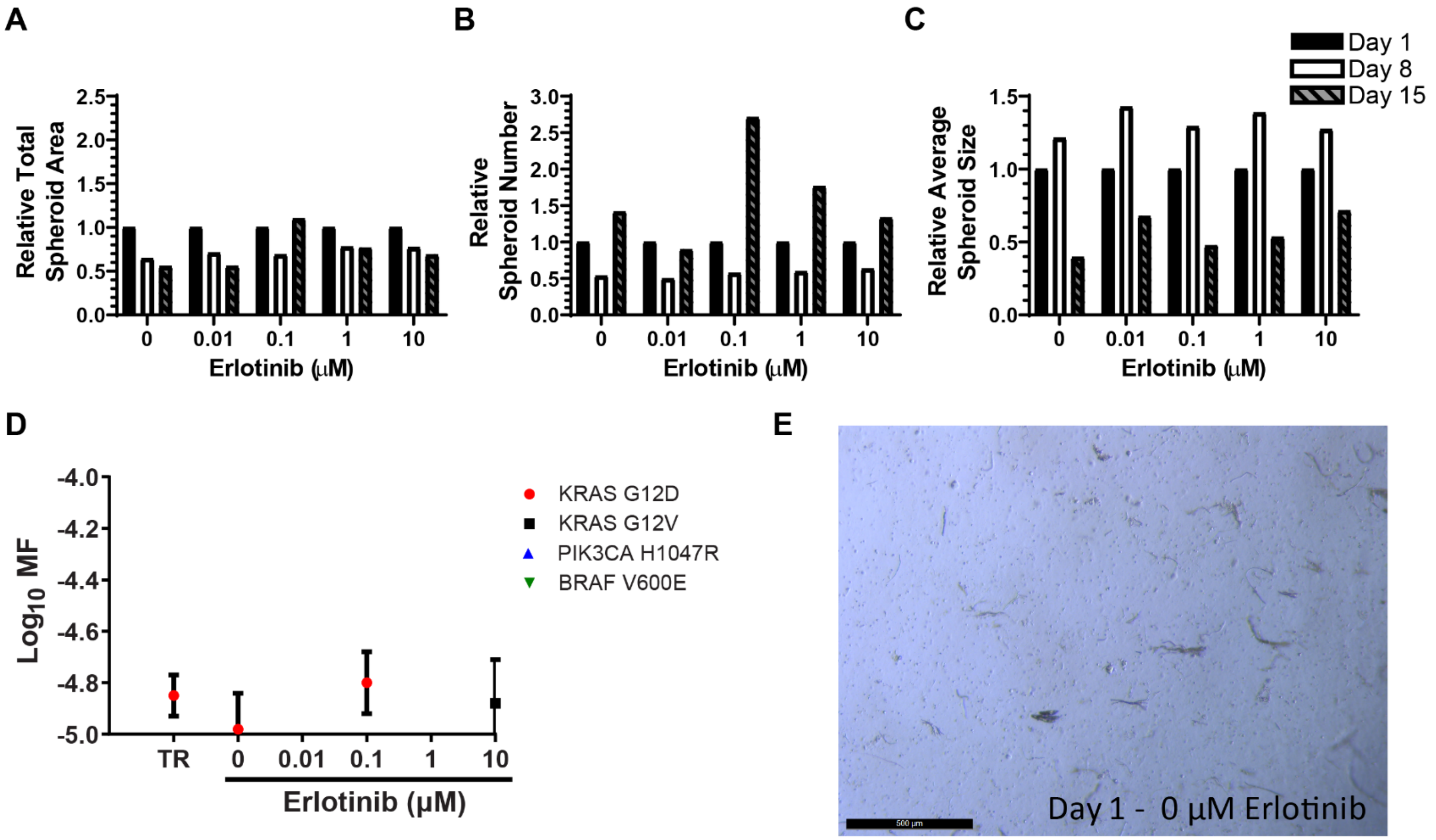

S9 Fig. Tumor 8.

Quantification of (A) relative total spheroid area, (B) relative spheroid number, and (C) relative average spheroid size. Quantified mutant subpopulations are plotted (D), with error bars indicating standard deviation. *KRAS* G12V MF measurements were not obtained for the 0 µM erlotinib culture. An example of spheroid culture appearance (E) is provided, in which the scale bar = 500 µm.
