# Supplementary material for: Outgrowth of erlotinib-resistant subpopulations recapitulated in patient-derived lung tumor spheroids and organoids
Source: PLoS One. 2020 Sep 8;15(9):e0238862. doi: 10.1371/journal.pone.0238862 (PMC7478813; doi:10.1371/journal.pone.0238862)
Supplement: S10 Fig — Quantification of (A) relative total spheroid area, (B) relative spheroid number, and (C) relative average spheroid size. Quantified mutant subpopulations are plotted (D), with error bars indicating standard deviation. The KRAS G12V mutant subpopulation quantified in the 0.01 and 0.1 μM erlotinib cultures were significantly greater than that of the 0 μM erlotinib culture or the Tumor 6 TR (one-tailed Mann Whitney test, P = 0.0500). The PIK3CA H1047R mutant subpopulation quantified in the 0.01 and 0.1 μM erlotinib cultures were significantly greater than those of the 0 μM erlotinib culture or the Tumor 6 TR (one-tailed Mann Whitney test, P = 0.0500). KRAS G12V MF measurements were not obtained for the 1 μM erlotinib culture. An example of spheroid culture appearance (E) is provided, in which the scale bar = 500 μm. (PDF) [file pone.0238862.s013.pdf]

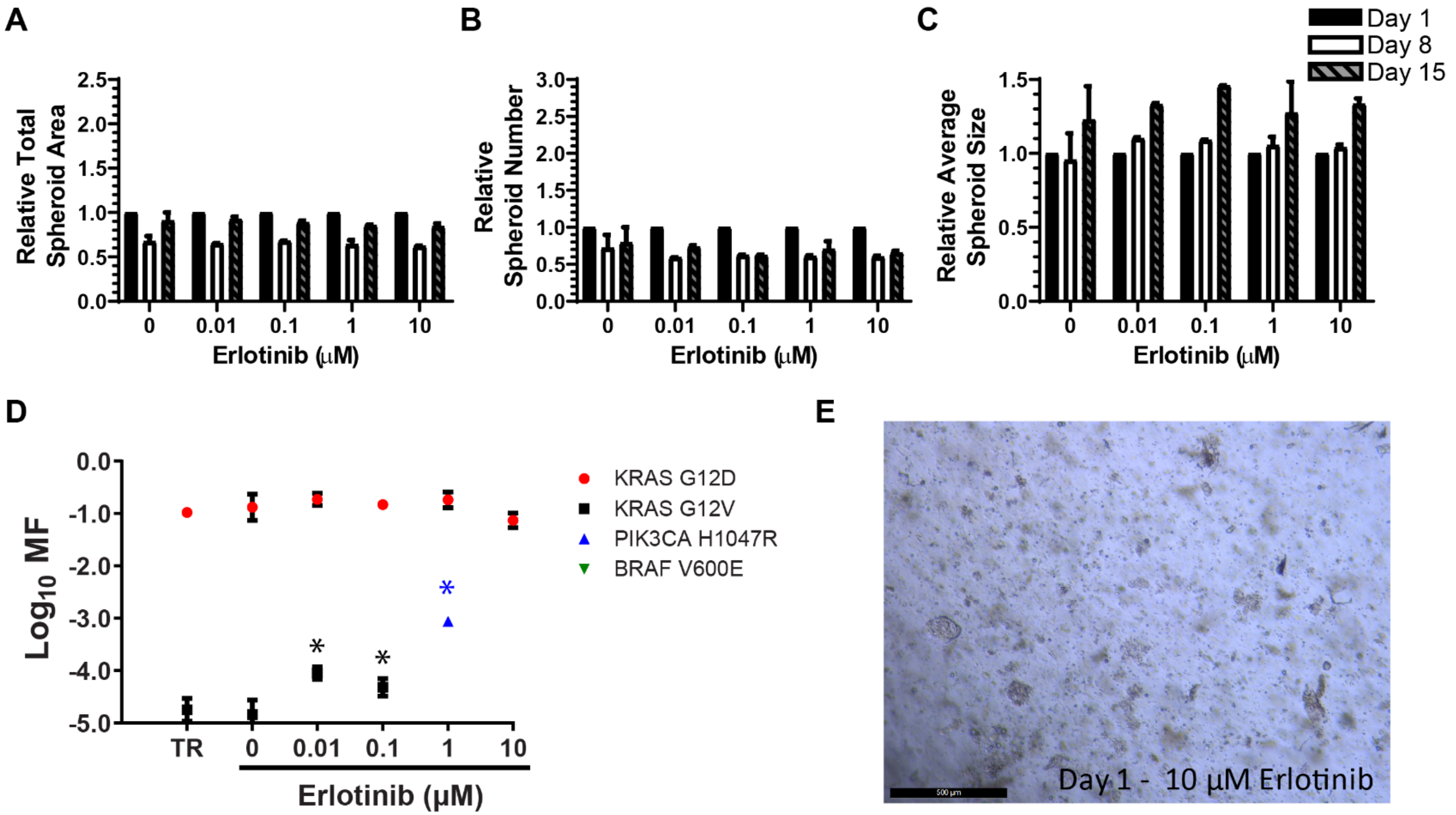

S10 Fig. Tumor 9.

Quantification of (A) relative total spheroid area, (B) relative spheroid number, and (C) relative average spheroid size. Quantified mutant subpopulations are plotted (D), with error bars indicating standard deviation. The *KRAS* G12V mutant subpopulation quantified in the 0.01 and 0.1 µM erlotinib cultures were significantly greater than that of the 0 µM erlotinib culture or the Tumor 6 TR (one-tailed Mann Whitney test, P = 0.0500). The *PIK3CA* H1047R mutant subpopulation quantified in the 0.01 and 0.1 µM erlotinib cultures were significantly greater than those of the 0 µM erlotinib culture or the Tumor 6 TR (one-tailed Mann Whitney test, P = 0.0500). *KRAS* G12V MF measurements were not obtained for the 1 µM erlotinib culture. An example of spheroid culture appearance (E) is provided, in which the scale bar = 500 µm.
