# Supplementary material for: Outgrowth of erlotinib-resistant subpopulations recapitulated in patient-derived lung tumor spheroids and organoids
Source: PLoS One. 2020 Sep 8;15(9):e0238862. doi: 10.1371/journal.pone.0238862 (PMC7478813; doi:10.1371/journal.pone.0238862)
Supplement: S11 Fig — Quantification of (A) relative total spheroid area, (B) relative spheroid number, and (C) relative average spheroid size, with error bars indicating the standard error of the mean. Quantified mutant subpopulations are plotted (D), with error bars indicating standard deviation. Three mutant subpopulations were detected and quantified, but no erlotinib-treated culture showed a significantly larger subpopulation than both the 0 μM erlotinib culture and the Tumor 10 TR (one-tailed Mann Whitney test, P = 0.0500). PIK3CA H1047R MF measurements were not obtained for the 0.1 or 1 μM erlotinib cultures. An example of spheroid culture appearance is provided (E), in which the scale bar = 200 μm. (PDF) [file pone.0238862.s014.pdf]

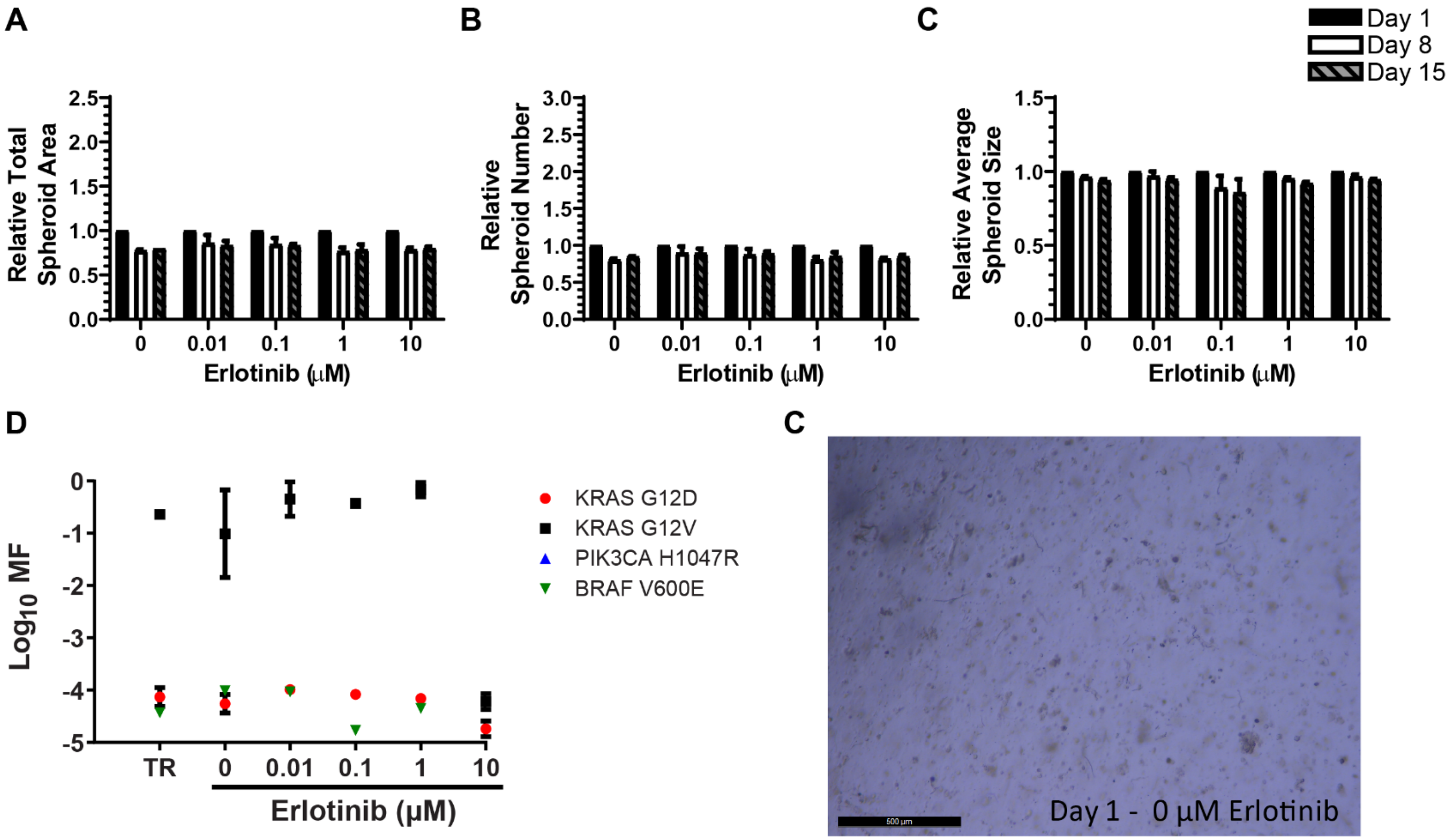

S11 Fig. Tumor 10.

Quantification of (A) relative total spheroid area, (B) relative spheroid number, and (C) relative average spheroid size, with error bars indicating the standard error of the mean. Quantified mutant subpopulations are plotted (D), with error bars indicating standard deviation. Three mutant subpopulations were detected and quantified, but no erlotinib-treated culture showed a significantly larger subpopulation than both the 0 μM erlotinib culture and the Tumor 10 TR (one-tailed Mann Whitney test, P = 0.0500). *PIK3CA* H1047R MF measurements were not obtained for the 0.1 or 1 μM erlotinib cultures. An example of spheroid culture appearance is provided (E), in which the scale bar = 200 μm.
