# Supplementary material for: Outgrowth of erlotinib-resistant subpopulations recapitulated in patient-derived lung tumor spheroids and organoids
Source: PLoS One. 2020 Sep 8;15(9):e0238862. doi: 10.1371/journal.pone.0238862 (PMC7478813; doi:10.1371/journal.pone.0238862)
Supplement: S13 Fig — (A) Total spheroid area, (B) spheroid number, and (C) average spheroid size measured at day 3 (0.43 weeks) and passage at Week 10 were set to one for each well and relative repeat measures over time are plotted. Measurements on Week 22 were considered unreliable and removed because inconsistent repeat measures were obtained for multiple tumors on this day. (PDF) [file pone.0238862.s016.pdf]

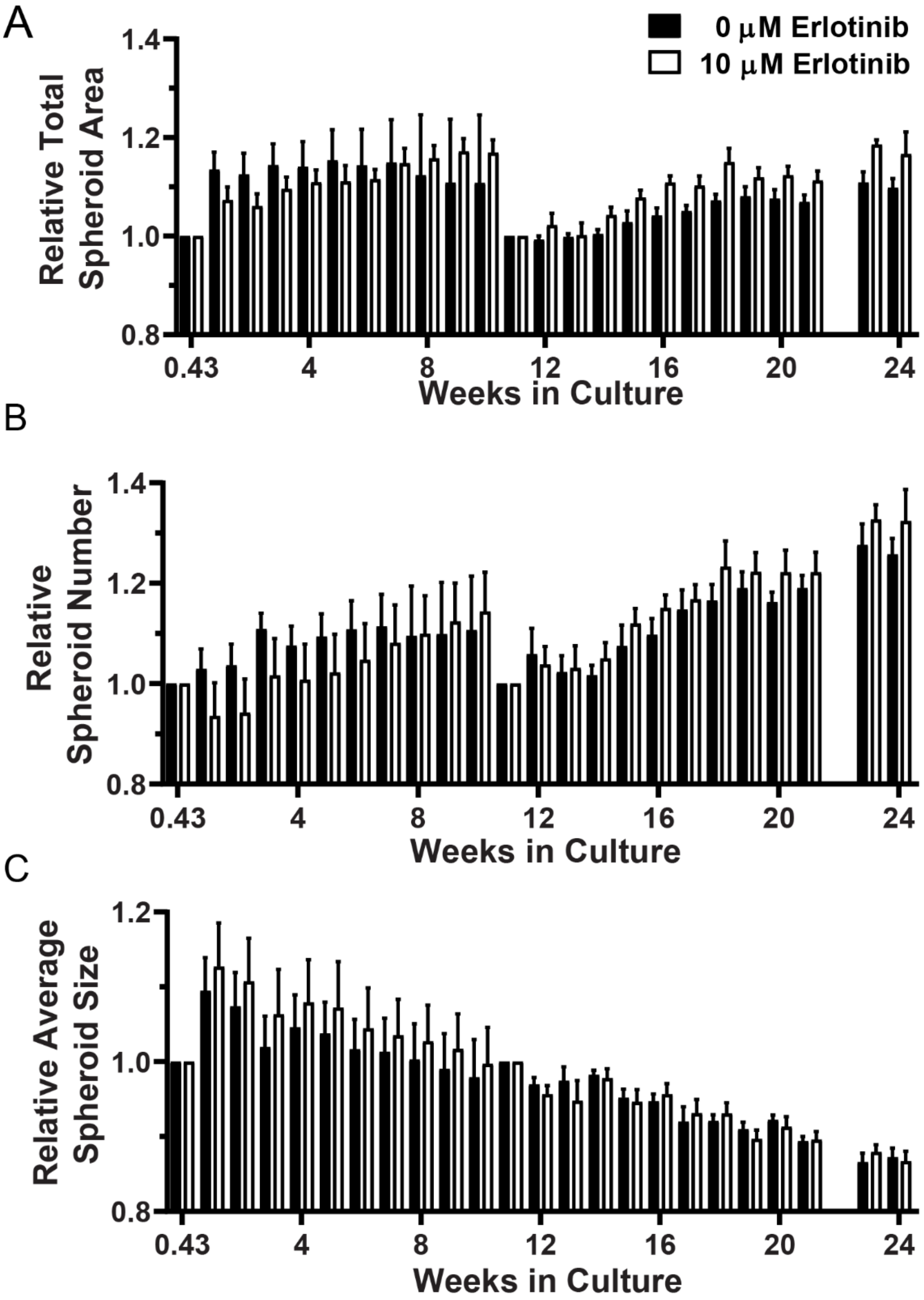

S13 Fig. Changes in Tumor 13 Spheroid Culture Parameters Over Time in Culture. (A) Total spheroid area, (B) spheroid number, and (C) average spheroid size measured at day 3 (0.43 weeks) and passage at Week 10 were set to one for each well and relative repeat measures over time are plotted. Measurements on Week 22 were considered unreliable and removed because inconsistent repeat measures were obtained for multiple tumors on this day.
