# Supplementary material for: Outgrowth of erlotinib-resistant subpopulations recapitulated in patient-derived lung tumor spheroids and organoids
Source: PLoS One. 2020 Sep 8;15(9):e0238862. doi: 10.1371/journal.pone.0238862 (PMC7478813; doi:10.1371/journal.pone.0238862)
Supplement: S14 Fig — The organoids shown were cultured from Tumor 12 (panel A), Tumor 13 (panels B and F), Tumor 14 (panels C, D and E). The scale bar in panels A, B, D, E and F is 100 μm. The scale bar in panel C is 200 μm. D and E show organoids that appear to have attached to the bottom of the plate and grown primarily as 2D cultures. (PDF) [file pone.0238862.s017.pdf]

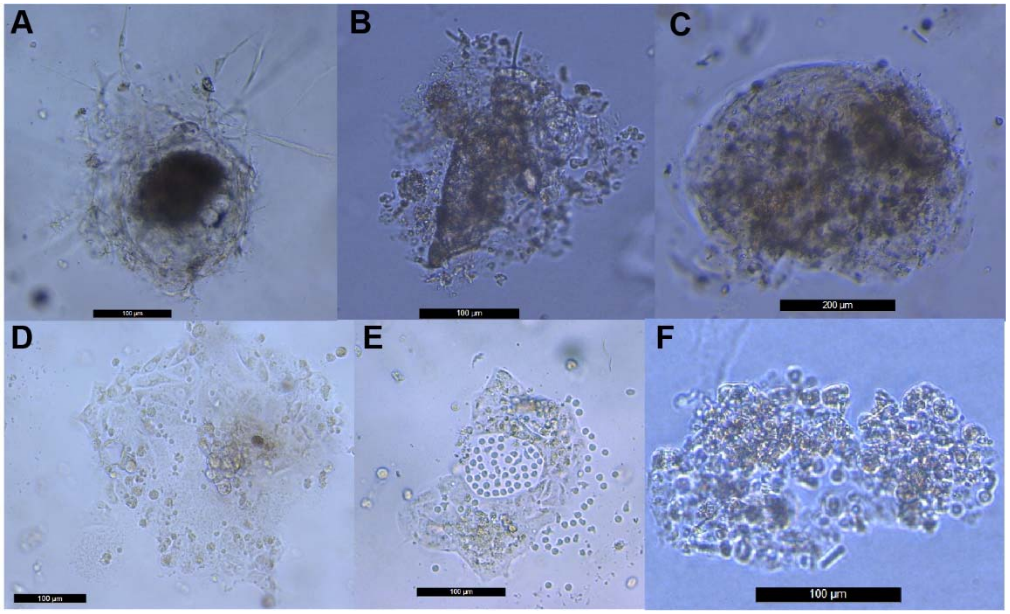

S14 Fig. Additional Examples of Organoid Morphologies. The organoids shown were cultured from Tumor 12 (panel A), Tumor 13 (panels B and F), Tumor 14 (panels C, D and E). The scale bar in panels A, B, D, E and F is 100 μm. The scale bar in panel C is 200 μm. D and E show organoids that appear to have attached to the bottom of the plate and grown primarily as 2D cultures.
